# HIV-1 Gp120 clade B/C induces a GRP78 driven cytoprotective mechanism in astrocytoma

**DOI:** 10.18632/oncotarget.19474

**Published:** 2017-07-22

**Authors:** Sheila N. López, Madeline Rodríguez-Valentín, Mariela Rivera, Maridaliz Rodríguez, Mohan Babu, Luis A. Cubano, Huangui Xiong, Guangdi Wang, Lilia Kucheryavykh, Nawal M. Boukli

**Affiliations:** ^1^ Biomedical Proteomics Facility, Department of Microbiology and Immunology, Universidad Central del Caribe, School of Medicine, Bayamón, PR, USA; ^2^ Department of Biochemistry, Research and Innovation Center, University of Regina, Saskatchewan, Canada; ^3^ Department of Pharmacology and Experimental Neuroscience, University of Nebraska Medical Center, Omaha, NE, USA; ^4^ RCMI Cancer Research Center, Xavier University of Louisiana, New Orleans, LA, USA; ^5^ Department of Biochemistry, Universidad Central del Caribe, School of Medicine, Bayamón, PR, USA

**Keywords:** HIV-1 clades B and C, Gp120, GRP78, quantitative proteomics (tandem mass tag), unfolded protein response (UPR)

## Abstract

HIV-1 clades are known to be one of the key factors implicated in modulating HIV-associated neurocognitive disorders. HIV-1 B and C clades account for the majority of HIV-1 infections, clade B being the most neuropathogenic. The mechanisms behind HIV-mediated neuropathogenesis remain the subject of active research. We hypothesized that HIV-1 gp120 clade B and C proteins may exert differential proliferation, cell survival and NeuroAIDS effects in human astrocytoma cells via the Unfolded Protein Response, an endoplasmic reticulum- based cytoprotective mechanism. The differential effect of gp120 clade B and C was evaluated using for the first time a Tandem Mass Tag isobaric labeling quantitative proteomic approach. Flow cytometry analyses were performed for cell cycle and cell death identification. Among the proteins differentiated by HIV-1 gp120 proteins figure cytoskeleton, oxidative stress, UPR markers and numerous glycolytic metabolism enzymes. Our results demonstrate that HIV-1 gp120 B induced migration, proliferative and protective responses granted by the expression of GRP78, while HIV-1 gp120 C induced the expression of key inflammatory and pro-apoptotic markers. These novel findings put forward the first evidence that GRP78 is a key player in HIV-1 clade B and C neuropathogenic discrepancies and can be used as a novel target for immunotherapies.

## INTRODUCTION

Approximately 40-60% of HIV-infected individuals in the United States develop cognitive and motor disorders also called HIV-associated neurocognitive disorders (HAND) [1–3] and this prevalence appears to be rising each year among HIV/AIDS patients [4]. HAND is an encephalopathy induced by HIV infection, and fueled by immune activation of macrophages and T-lymphocytes [5]. These cells are actively infected with HIV-1 and secrete neurotoxins of both host and viral origin affecting brain cells such as astrocytes and neurons. The affected astrocytes that normally nurture and protect neurons may now end up harming these cells. HIV-1 invades the Central Nervous System (CNS) at early stage after primary infection [6] which may result ultimately in the development of several types of neurological disorders. Studies have shown that HIV-1 Tat and gp120 proteins are neurotoxic and have been suggested as one of the contributing factors of HIV-1 associated-dementia [7–10].

Despite the success of combined antiretroviral therapy to lower viral replication it still cannot provide complete protection from HIV-1 induced neuronal injury and there is no effective treatment for HAND at present. Another obstacle concerning treatment is the genetic diversity of HIV-1, specially, with regard to the expansion of distinct viral clades (from A to K) in different geographical regions, clade B being the most predominant in Europe and America, while clade C exists mainly in sub-saharan Africa and Asia [11]. HIV-1 clade C is responsible for more than half of new HIV-1 infections and is likely to affect the United States in the near future due to global travel [12]. HIV clade B infected patients develop HAND with a higher incidence as compared to HIV clade C infected subjects, suggesting clade-specific differences in neuropathogenicity [13]. Clade specific studies have mainly focused on HIV-1 Tat, and have strongly suggested that these clade differences are important determinants of HAND pathogenesis [14]. However, the underlying mechanism responsible to exert differential effects in HAND development between HIV-1 gp120 clade B and C gp120 remains poorly understood.

The N-glycosylated HIV-1 envelope protein gp120 is known to be a key player in HIV neuropathogenesis. Gp120 causes direct neurotoxicity and cytokine and chemokine modulation in microglia and astrocytes [15, 16]. Gp120 interplay with cell surface receptors, particularly CD4, CCR5 and CXCR4, is crucial for virus entry into cells. Astrocytes lack CD4 receptor under normal conditions but can still bind to gp120 by CXCR4 and CCR5 chemokine receptors [17, 18]. The astrocytic dysfunction and its effect on HIV-1 neurotoxicity has been shown to contribute to HAND pathogenesis and reported as a potential biomarker for motor and cognitive disorder [1, 6, 19].

More recently, Zayyad et al showed that HIV invasion into CNS is thought to occur through the “Trojan horse hypothesis”[20]. This model states that an infected cell can get to the CNS by the disruption of the blood brain barrier integrity allowing CD4+ T cells and HIV-1 infected macrophages to enter the CNS [21]. Release of HIV-1 virions from peripheral infected immune cells can contribute to microglial and astrocytic infection while neurons remain susceptible to damage by viral toxins secretion’s (such as Tat and gp120) from activated or infected macrophages, microglia and astrocytic cells. CNS immune activation releases neurotoxic mediators such as pro-inflammatory cytokines, glutamate and reactive nitrogen and oxidative species (RNS, ROS), which consequently causes neuronal dysfunction, dendritic loss and neuronal death [22]. Release of viral proteins will therefore enhance the inflammatory processes that potentiate neuropathogenesis [23].

Recent studies demonstrated that oxidative stress plays a role in the progression of acquired immunodeficiency syndrome (AIDS) leading patients to HIV associated dementia [24]. Neurotoxicity induced by HIV-1 Tat and gp120 proteins may lead to oxidative stress [25] triggering the endoplasmic reticulum (ER) stress response [26]. The ER plays an important role in the regulation of proteins and cell homeostasis, serving as a crucial organelle when stressors such as drugs and HIV enter the system. Accumulation of misfolded proteins in the ER can disrupt ER function resulting in ‘ER stress’. The ER responds by triggering specific signaling pathways including the Unfolded Protein Response (UPR) [27, 28]. The accumulation of misfolded proteins activates three ER stress receptors: Protein RNA Kinase-like Endoplasmic Reticulum kinase (PERK), Inositol Requiring Enzyme 1alpha (IRE1α) and Activating transcription factor 6 (ATF6) in charge of activating three different pathways to respond to ER stress in the cell [29]. UPR is activated when chaperone Glucose Regulating Protein 78 kDa (GRP78) detaches itself from UPR receptors to help refold misfolded proteins accumulated in the ER. There is evidence of the multiple roles of GRP78 in different cellular localizations besides the ER, such as the mitochondria, cytoplasm, nucleus and more recently the plasma membrane [30, 31]. Depending on localization, GRP78 can act as a regulator in cell survival, proliferation, inflammation, apoptosis and immunity as well as several other key cellular functions [30–33]. ER stress can induce GRP78 re-localization from the ER to the plasma membrane and can act as a growth signal and as an immuno-recognition molecule where it binds to the major histocompatibility complex I and contribute to viral entry [33–35]. Despite what is already known about GRP78, its mechanism on regulation of HIV-1 pathogenesis is still unknown.

The current study allowed the identification of key differentially expressed proteins between treated gp120 clade B and C in astrocytoma cells. Among the significantly up-regulated proteins by HIV-1 gp120 B and C treatments figure structural, cytoskeleton, endoplasmic reticulum and oxidative stress markers, inflammatory mediators, autophagy and metabolic factors. Our findings establish that HIV-1 gp120 clade B had a higher migration rate, increased proliferation and a protective response as compared to gp120 C treated astrocytoma. Moreover, the results presented here, show for the first time, that HIV-1 gp120 clade B protein induced the expression of a key ER-stress chaperone, GRP78. We hypothesized that the overexpression of GRP78 by HIV-1 gp120 clade B may protect astrocytoma cells from ER stress and oxidative injury that may lead to NeuroAIDS. This observation is based on the fact that HIV-1 gp120 clade B induced a more protective and proliferative response in astrocytoma cells as compared to HIV-1 gp120 clade C that lacked GRP78 expression and proliferative capacity. Overall, this study provides an insight on how HIV-1 gp120 clades B and C differ in neuropathogenesis by providing the basis for the development of new complementary therapeutic approaches to treat HAND.

## RESULTS

### HIV-1 gp120 clade B and C induced a differential interclade effect on cell viability and cell death in U87-MG cells

**Figure 1:**
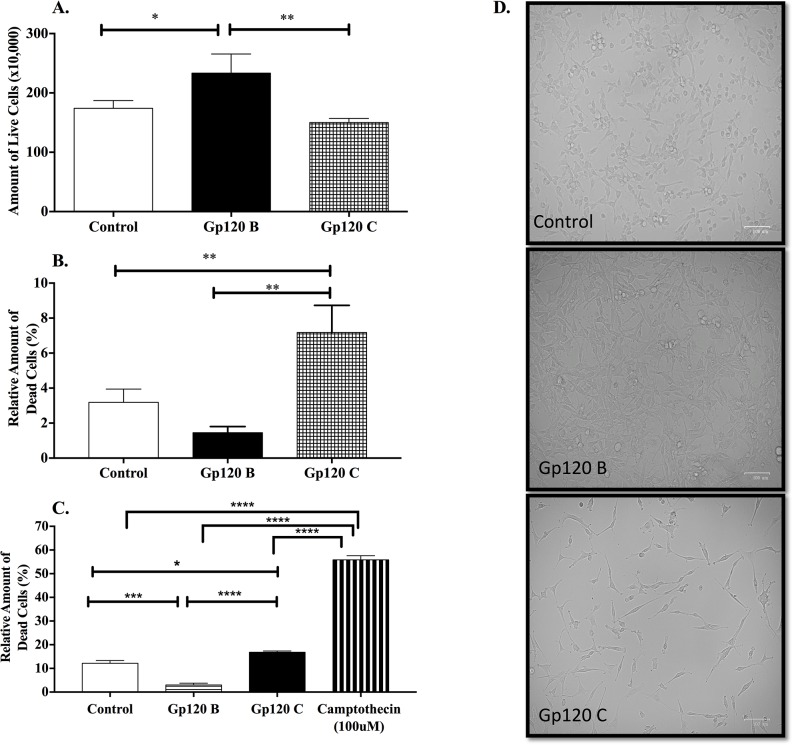
HIV-1 clade B and C gp120 proteins induced a differential interclade effect in cell viability and cell death in U87-MG cells Experiments were performed for control U87-MG vehicle (medium only) and cells treated with gp120 from clade B and C for 24 hours. Live and dead cells were counted with use of trypan blue staining. **(A)** Cell viability was evaluated as a total amount of live cells. **(B)** Relative amount of dead cells was evaluated as percent equivalent to the total amount of cells. **(C)** Cell death percentage measured by propidium iodide flow cytometry analysis after HIV-1 gp120 B and C treatments. **(D)** Cell density bright-field micrographs at 100um magnification with an adjusted +20% brightness for control and gp120 C and +40% contrast for gp120 B. Mean ± SEM and statistical significance was determined using one-way ANOVA, P ≤ 0.05 (N=3).

Trypan blue cell viability assay was used for U87-MG cells to determine the amount of live and dead cells after gp120 clade B and clade C treatment in astrocytoma. As shown in Figure 1, astrocytoma cells treated with HIV-1 gp120 clade B showed an increased amount of live cells when compared to control (untreated) cells (233.3 ± 18.67 vs. 174 ± 7.64). The amount of live cells in HIV-1 gp120 clade C treated group was lower when compared to HIV-1 gp120 clade B treated cells (150 ± 4.04 vs. 233.3 ± 18.67) but no statistical difference was observed in the amount of cells between control and HIV-1 gp120 clade C, as shown in Figure 1A and in the cell treatment micrographs presented in Figure 1D. The relative amount of dead cells was lower after HIV-1 gp120 clade B treatment when compared to HIV-1 gp120 clade C treated cells which showed nearly 7% of cell death (1.46% ± 0.20 vs. 7.18% ± 0.89) (Figure 1B).

To further validate the effect of HIV-1 gp120 clade B and clade C in cell viability, the amount of cell death was subsequently measured using propidium iodide flow cytometry analysis. Figure 1C depicts that the relative amount of dead cells increased when cells were treated with gp120 C as compared to control (16.85% ± 0.35 vs 12.15% ± 0.85) and gp120 clade B (2.98% ± 0.45). Camptothecin was used as a positive control for cell death. Gp120 clades B and C-induced a differential effect on viability and cell death when A172, a glioblastoma cell line, was treated with gp120 proteins for 24 hours (Supplementary Figure 1). Our results imply that HIV-1 gp120 clade C treated cells induced a cytotoxic effect in the cell while gp120 clade B induced an anti-apoptotic response.

### Isobaric labeling tandem mass tag quantitative proteomic profiling revealed a differential protein expression in U87-MG cells treated with HIV-1 gp120 clade B and C

**Figure 2:**
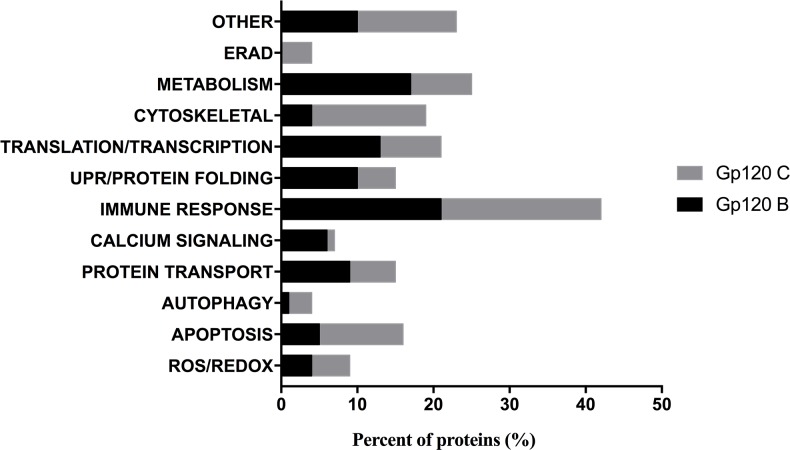
Distribution of TMT labeled proteins after HIV-1 gp120 B and C treatment as identified by peptide mass fingerprinting and organized according to biological processes Identified proteins by MASCOT database were distributed and analyzed by GSEA according to their biological function in in HIV-1 gp120 clade B and C proteins (200ng/ml) treated U87-MG cells.

The differential expression of proteins between vehicle (medium only) and treated (200 ng/ml of HIV-1 gp120 B/C) U87-MG cells was determined based on an isobaric labeling TMT quantitative proteomic approach. In total, 158 proteins were identified from control and treated U87-MG cell lysates. Among them, 72 significantly differentially expressed proteins were identified for HIV-1 gp120 clade B treated cells and 86 for HIV-1 gp120 clade C. Identified proteins in HIV-1 clade B and C treated cells were organized according to biological processes and relevant functional pathways (Figure 2) and (Tables 1 and 2). Proteins related to apoptosis, immune response, UPR, ROS and ERAD with p-values ≤ 0.025 (P-value adjusted as a false discovery rate, FDR) were selected for HIV-1 gp120 clade B (Table 1) and HIV-1 gp120 clade C (Table 2) treated cells. Supplementary Tables 1 and 2 showed additional differentially expressed proteins in other altered biological and cellular pathways induced by HIV-1 gp120 clade B and C treated astrocytoma, respectively.

**Table 1:** Differentially expressed proteins in HIV-1 gp120 B treated astrocytoma. Classification of differentially expressed proteins were distributed according to their biological function and analyzed by GSEA. Gene ontology identification (GO.ID), Sum of identified peptides (Σ# Peptides), Sum of the numbers of peptide spectrum matches (Σ# PSM’s), Identified amino acid numbers (# AA’s), Molecular weight (MW, kDa), Calculated isoelectric point (pH, calc. PI) and Adjusted P-value as false discovery rate (FDR). Proteins with a FDR ≤ 0.025 were selected as significant for Gp120 B treated cells

Gp120 B Induced Proteins									
GO.ID [a]	GSEA assigned Biological Process	Σ# Peptides [b]	Σ# PSM’s [c]	AA’s # [d]	MW [e]	calc. PI [f]	Adjusted P-value (FDR) [g]	Gene	Description
**Apoptosis/DNA Damage**									
REAC:75153	apoptotic execution phase	2	6	4684	531.5	5.96	0.018	PLEC	Plectin
		1	1	213	21.4	10.93		HIST1H1C	Histone H1.2
		2	4	2452	282.1	5.34		SPTAN1	Spectrin alpha chain
GO:0008656	cysteine-type endopeptidase activator activity involved in apoptotic process	1	2	83	8.8	4.42	0.012	GNB2L1	Receptor of activated protein C kinase 1
GO:2000210	positive regulation of anoikis	1	1	179	19.2	8.73	0.032	PTRH2	Peptidyl-tRNA hydrolase 2, mitochondrial
**ROS/Redox**									
GO:1903204	negative regulation of oxidative stress-induced neuron death	1	2	83	8.8	4.42	0.007	GNB2L1	Receptor of activated protein C kinase 1
GO:0030235	nitric-oxide synthase regulator activity	5	8	724	83.2	5.03	0.018	HSP90AB1	Heat shock protein HSP 90-beta
GO:0016620	oxidoreductase activity, acting on the aldehyde or oxo group of donors, NAD or NADP as acceptor	1	3	151	16.9	8.65	0.001	ALDH1A3	Aldehyde dehydrogenase family 1 member A3
		6	30	335	36	8.46		GAPDH	Glyceraldehyde-3-phosphate dehydrogenase
**Immune Response**									
REAC:983170	antigen Presentation: Folding, assembly and peptide loading of class I MHC	2	2	654	72.3	5.16	0.001	HSPA5	78 kDa glucose-regulated protein
		1	3	279	31.9	7.37		PDIA3	Protein disulfide-isomerase A3
		1	3	296	34.7	4.34		CALR	Calreticulin
KEGG:04612	antigen processing and presentation	5	8	724	83.2	5.03	0.003	HSP90AB1	Heat shock protein HSP 90-beta
GO:0002479	antigen processing and presentation of exogenous peptide antigen via MHC class I, TAP-dependent	1	3	149	16.8	6.58	0.011	UBC	Polyubiquitin-C
REAC:1169410	antiviral mechanism by IFN-stimulated genes	2	4	1403	154.7	5.16	0.042	EIF4G1	Eukaryotic translation initiation factor 4 gamma 1
		1	3	149	16.8	6.58		UBC	Polyubiquitin-C
		2	6	200	23.2	5.69		EIF4A1	Eukaryotic initiation factor 4A-I
REAC:3134963	DEx/H-box helicases activate type I IFN and inflammatory cytokines production	1	3	599	67.4	5.82	0.025	DHX9	ATP-dependent RNA helicase A
REAC:1236974	ER-Phagosome pathway	1	3	279	31.9	7.37	0.031	PDIA3	Protein disulfide-isomerase A3
		1	3	149	16.8	6.58		UBC	Polyubiquitin-C
GO:0075733	intracellular transport of virus	1	2	103	11.7	10.33	0.024	RAN	GTP-binding nuclear protein Ran
GO:0042288	MHC class I protein binding	2	7	518	55.3	5.4	0.005	ATP5B	ATP synthase subunit beta, mitochondrial
		2	15	372	41.7	4.91		TUBB	Tubulin beta-3 chain
GO:0023026	MHC class II protein complex binding	8	31	516	56.2	8.44	0.000	PKM	Pyruvate kinase PKM
		5	8	724	83.2	5.03		HSP90AB1	Heat shock protein HSP 90-beta
		3	9	233	26.5	4.87		YWHAE	14-3-3 protein epsilon
GO:0032481	positive regulation of type I interferon production	1	1	557	64	6.83	0.013	XRCC6	X-ray repair cross-complementing protein 6
GO:0046598	positive regulation of viral entry into host cell	2	16	135	14.7	5.5	0.045	LGALS1	Galectin-1
GO:0010803	regulation of tumor necrosis factor-mediated signaling pathway	1	2	83	8.8	4.42	0.041	GNB2L1	Receptor of activated protein C kinase 1
**UPR/Protein Folding**									
GO:0036500	ATF6-mediated unfolded protein response	2	2	654	72.3	5.16	0.002	HSPA5	78 kDa glucose-regulated protein
		1	3	296	34.7	4.34		CALR	Calreticulin
GO:0051084	‘de novo’ posttranslational protein folding	2	7	362	40.5	4.89	0.001	TUBB4A	Tubulin beta-4A chain
		1	1	389	42.3	7.61		CCT4	T-complex protein 1 subunit delta
		1	1	84	10.1	4.63		TBCA	Tubulin-specific chaperone A
GO:0001948	glycoprotein binding	1	2	1037	115.1	6.57	0.000	ITGA3	Integrin alpha-3
		2	4	2315	245.7	5.97		FLNA	Filamin-A
REAC:381070	IRE1alpha activates chaperones	2	2	2429	257.9	6.49	0.029	TLN1	Talin-1
GO:0017166	vinculin binding	2	2	2429	257.9	6.49	0.022	TLN1	Talin-1
**Other**									
GO:1902808	positive regulation of cell cycle G1/S phase transition	1	1	560	62.7	9.68	0.003	CDC6	Cell division control protein 6 homolog
		1	1	112	12	9.61		PHB2	Prohibitin-2

[a] Gene ontology identification

[b] Sum of identified peptides

[c] Sum of the numbers of peptide spectrum matches

[d] Identified aminoacid numbers

[e] Molecular weight, kDa

[f] Calculated isoelectric point, pH

[g] Adjusted P-value as false discovery rate

**Table 2:** Differentially expressed proteins in HIV-1 gp120 C treated astrocytoma Classification of differentially expressed proteins were distributed according to their biological function and analyzed by GSEA. Gene ontology identification (GO.ID), Sum of identified peptides (Σ# Peptides), Sum of the numbers of peptide spectrum matches (Σ# PSM’s), Identified amino acid numbers (# AA’s), Molecular weight (MW, kDa), Calculated isoelectric point (pH, calc. PI) and Adjusted P-value as false discovery rate (FDR). Proteins with a FDR ≤ 0.025 were selected as significant for Gp120 C treated cells

Gp120 C Induced Proteins									
GO.ID [a]	GSEA assigned Biological Process	Σ# Peptides [b]	Σ# PSM’s [c]	AA’s # [d]	MW [e]	calc. PI [f]	Adjusted P-value (FDR) [g]	Gene	Description
**ROS/Redox**									
GO:0016209	antioxidant activity	1	2	208	23.7	7.17	0.004	GSTO1	Glutathione S-transferase omega-1
REAC:3299685	detoxification of Reactive Oxygen Species	1	7	279	32.1	8.51	0.001	P4HB	Protein disulfide-isomerase
		1	1	224	25	6.38	PRDX6	Preoxiredoxin-6
		1	1	125	12.9	9.41	PRDX5	Peroxiredoxin-5
GO:0016860	intramolecular oxidoreductase activity	1	3	115	12.5	7.88	0.002	MIF	Macrophage migration inhibitory factor
		3	5	213	22.9	6.92	TPI1	Triosephosphate isomerase
GO:1902175	regulation of oxidative stress-induced intrinsic apoptotic signaling pathway	1	1	669	72.2	9.23	0.005	SFPQ	Splicing factor, proline- and glutamine-rich
**Apoptosis/DNA Damage**									
REAC:111465	apoptotic cleavage of cellular proteins	10	55	431	49.6	5.25	0.004	VIM	Vimentin
		2	5	465	53.2	6.52	LMNA	Prelamin-A/C
REAC:111447	activation of BAD and translocation to mitochondria	2	5	210	23.8	4.88	0.002	YWHAQ	14-3-3 protein theta
		2	6	125	14	4.41	YWHAZ	14-3-3 protein zeta/delta
GO:1902166	negative regulation of intrinsic apoptotic signaling pathway in response to DNA damage by p53 class mediator	1	1	181	19.6	4.55	0.003	CD44	CD44 antigen
		1	3	115	12.5	7.88	MIF	Macrophage migration inhibitory factor
REAC:2559586	DNA Damage/Telomere Stress Induced Senescence	2	13	126	13.9	10.32	0.013	HIST2H2BE	Histone H2B type 2-E
		2	6	103	11.4	11.36	HIST4H4	Histone H4
GO:2000210	positive regulation of anoikis	1	2	603	67.5	9.72	0.021	MYBBP1A	Myb-binding protein 1A
		REAC:69473	G2/M DNA damage checkpoint	2	5	210	23.8	4.88	0.000	YWHAQ	14-3-3 protein theta
2	13			126	13.9	10.32	HIST2H2BE	Histone H2B type 2-E
2	6			103	11.4	11.36	HIST4H4	Histone H4
2	6	125	14	4.41	YWHAZ	14-3-3 protein zeta/delta
**ERAD**									
REAC:5362768	Hh mutants that don’t undergo autocatalytic processing are degraded by ERAD	1	3	261	29.5	7.72	0.000	PSMA4	Proteasome subunit alpha type-4
		1	5	85	9.3	4.41	PSMA6	Proteasome subunit alpha type-6
		1	2	241	26.4	4.79	PSMA5	Proteasome subunit alpha type-5
		1	4	138	16	6.14	PSMD6	Proteosome subunit delta type-6
		1	1	591	64.5	6.86	PSMD2	Proteosome subunit delta type-2
**Immune Response**									
REAC:1169091	activation of NF-kappaB in B cells	1	3	261	29.5	7.72	0.000	PSMA4	Proteasome subunit alpha type-4
		1	5	85	9.3	4.41	PSMA6	Proteasome subunit alpha type-6
		1	2	241	26.4	4.79	PSMA5	Proteasome subunit alpha type-5
		1	4	138	16	6.14	PSMD6	Proteosome subunit delta type-6
		1	1	591	64.5	6.86	PSMD2	Proteosome subunit delta type-2
GO:0042056	chemoattractant activity	1	1	233	24.1	9.39	0.018	LGALS3	Galectin-3
		1	3	115	12.5	7.88	MIF	Macrophage migration inhibitory factor
REAC:3270619	IRF3-mediated induction of type I IFN	1	1	732	82.7	5.81	0.002	XRCC5	X-ray repair cross-complementing protein 5
		2	2	4097	465.2	7.17	PRKDC	DNA-dependent protein kinase catalytic subunit
GO:0023026	MHC class II protein complex binding	6	10	732	84.6	5.02	0.000	HSP90AA1	Heat shock protein HSP 90-alpha
		8	23	531	58	7.71	PKM	Pyruvate kinase PKM
		2	4	312	34.8	7.53	HSPA8	Heat shock cognate 71 kDa protein
GO:0038061	NIK/NF-kappaB signaling	1	3	261	29.5	7.72	0.000	PSMA4	Proteasome subunit alpha type-4
		1	5	85	9.3	4.41	PSMA6	Proteasome subunit alpha type-6
		1	2	241	26.4	4.79	PSMA5	Proteasome subunit alpha type-5
		1	4	138	16	6.14	PSMD6	Proteosome subunit delta type-6
		1	1	591	64.5	6.86	PSMD2	Proteosome subunit delta type-2
		4	16	692	79.9	5.17	ACTN4	Alpha actinin 4
GO:0044794	positive regulation by host of viral process	2	4	216	23.7	9.41	0.001	PPIB	Peptidyl-prolyl cis-trans isomerase B
		1	2	122	13.9	8.72	CFL1	Cofilin-1
GO:0032481	positive regulation of type I interferon production	2	4	517	55	5.6	0.006	HSPD1	60 kDa heat shock protein, mitochondrial
REAC:192823	viral mRNA Translation	1	2	64	7.3	11.65	0.005	RPL6	60S ribosomal protein L6
		1	1	180	19.8	9.95	RPL3	60S ribosomal protein L3
		1	2	115	11.7	4.54	RPLP2	60S ribosomal protein L2
		1	3	122	14	10.05	RPS4X	40S ribosomal protein S4, X isoform
**Protein Folding**									
GO:0051131	chaperone-mediated protein complex assembly	6	10	732	84.6	5.02	0.000	HSP90AA1	Heat shock protein HSP 90-alpha
		2	4	517	55	5.6	HSPD1	60 kDa heat shock protein, mitochondria
		1	2	474	50.9	6.8	CCT2	T-complex protein 1 subunit beta
GO:0006458	‘de novo’ protein folding	9	44	375	41.7	5.48	0.000	ACTB	Actin, cytoplasmic 1
		2	8	247	27.5	5.2	TUBA1B	Tubulin alpha-1B chain
		1	2	283	32	8.5	CCT5	T-complex protein 1 subunit epsilon
**Other**									
REAC:977225	amyloid fiber formation	2	13	126	13.9	10.32	0.020	HIST2H2BE	Histone H2B type 2-E
		2	6	103	11.4	11.36	HIST4H4	Histone H4

[a] Gene ontology identification

[b] Sum of identified peptides

[c] Sum of the numbers of peptide spectrum matches

[d] Identified aminoacid numbers

[e] Molecular weight, kDa

[f] Calculated isoelectric point, pH

[g] Adjusted P-value as false discovery rate

Among the differentially expressed proteins, figure proteins involved in redox reactions, such as glutathione S-transferase omega-1 (GSTO1), peroxiredoxin and protein disulfide isomerases that were identified in HIV-1 gp120 clade C treated cells. Gp120 clade B treated cells over-expressed heat shock protein (HSP 90-beta) and receptor activated protein C kinase 1, proteins that modulate nitric oxide synthase activity and oxidative-induced cell death, respectively. UPR markers, such as 78 kDa Glucose-Regulated Protein (GRP78), calreticulin, as well as autophagy marker lysosome-associated membrane glycoprotein 2 were up-regulated after HIV-1 gp120 clade B treatment, while HIV-1 gp120 clade C failed to induce these markers’ expression. Redox reactions have been shown to be tightly involved with UPR signaling by regulating ER homeostasis and to determine cellular fate to survival or death by controlling the switch from adaptive or fatal UPR signaling [36].

Additionally, cell cycle related markers were identified as up-regulated, such as Cell Division Control protein 6 homolog (CDC6), a positive regulator of cell cycle G1/S phase transition in HIV-1 gp120 clade B. G2/M DNA damage checkpoint markers such as, 14-3-3 protein zeta/delta, Histone H2B type 2-E and 14-3-3 protein theta (YWHAZ, HIST2H2BE and YWHAQ respectively) were up-regulated in HIV-1 gp120 clade C treated U87-MG cells. Moreover, proteins with known proliferative and chemoattractant activity such as, complement component 1 Q subcomponent-binding protein (C1QBP) and calreticulin (CALR) were uniquely up regulated by HIV-1 gp120 clade B, while galectin-3 (LGALS3) and macrophages migration inhibitory factor (MIF) were up-regulated specifically by HIV-1 gp120 clade C (Table 1, Table 2 and Supplementary Table 1) [37, 38]. Additionally, several cytoskeletal proteins were differentially expressed such as fascin, myosin 9, alpha actinin 4, and vimentin in HIV-1 gp120 clade C treated cells (Supplementary Table 1 and Supplementary Table 2). Interestingly, 10.9% of differentially expressed proteins were associated with apoptosis in HIV-1 gp120 clade C treated cells as compared to HIV-1 gp120 clade B treated cells (5.3%), suggesting that HIV-1 gp120 clade C induced a programmed cell death response with cell cycle specific perturbations.

Notably, HIV-1 gp120 clade C treated cells demonstrated up-regulation of several proteosomal subunits involved in the Endoplasmic Reticulum Associated Protein Degradation process (ERAD), know as a fight back mechanism to target misfolded proteins for degradation. Finally, one of the biological processes that was highly altered by HIV-1 gp120 proteins was the immune response. Major histocompatibility complex I and II related proteins, tumor necrosis factor (TNF), interferon signaling were up-regulated by HIV-1 gp120 clade B, while HIV-1 gp120 clade C induced the expression of proteins involved in NF-kB signaling, indicating that HIV-1 gp120 proteins activate the immuno-recognition response in astrocytoma.

### HIV-1 gp120 clade B stimulates cell proliferation and migration while HIV-1 gp120 clade C induces G0/G1 cell cycle arrest

**Figure 3:**
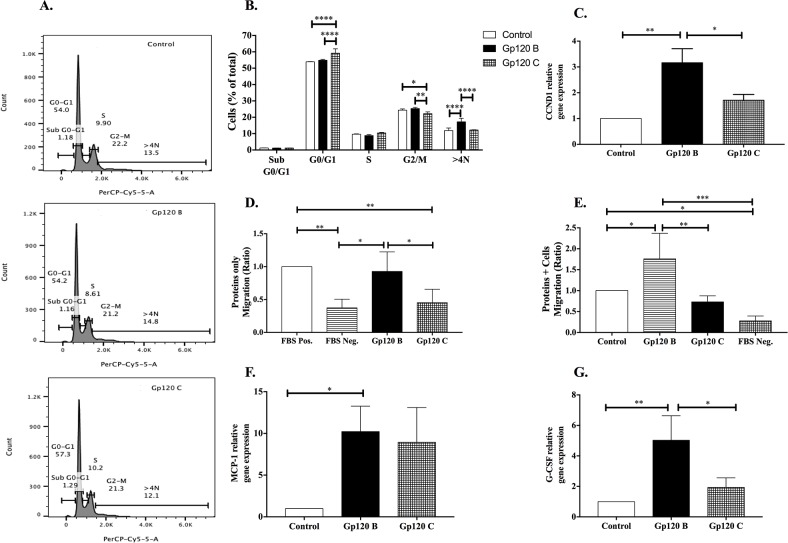
HIV-1 gp120 B stimulates cell proliferation and migration while HIV-1 gp120 C induces G0/G1 cell cycle arrest Flow cytometry analysis with use of 7-aminoactinomycin D staining and PerCP-Cy5-5-A filter set was used for the identification of cell cycle. The percentage of cells in G0/G1, S, and G2/M phases was identified based on DNA content. **(A)** Representative histogram images for cell cycle analysis after gp120 B and C treatments. **(B)** The graph represents the total distribution of cells at different phases of the cell cycle. Amount of cells at each phase is shown as a percentage of the total amount of cells. **(C)** Cyclin D1 mitogenic factor relative gene expression normalized to GAPDH. Migration assays were performed for 5 hours for HIV-1 gp120 clade B and C proteins alone **(D)** and astrocytoma cells treated with HIV-1 gp120 B and C proteins **(E)**. The total number of HMC3 migrated to the lower compartment was counted. Relative gene expression of migration and proliferative inducing-chemokine MCP-1 **(F)** and cytokine G-CSF **(G)** was determined after gp120 B and C 24 hours treated astrocytoma and normalized to GAPDH. Cells with FBS-free medium were used as a negative control for migration. Mean ± SEM and statistical significance was determined using one-way and two-way ANOVA as appropriate, P ≤ 0.05 (N=3).

Since the quantitative proteomics data in astrocytoma revealed altered protein expression in cell cycle regulation (Tables 1 and 2), the effect of HIV-1 gp120 clades B and C on cell cycle and mitosis was further validated by flow cytometry. U87-MG cells treated with HIV-1 gp120 clade C for 24 hours increased in G0/G1 cell cycle phase when compared to control (59.20% ± 1.90 vs. 53.90% ± 0.10) and to HIV-1 gp120 clade B treated cells (54.83% ± 0.32, Figure 3B). A similarly effect was observed with HIV-1 gp120 clade C treatment, causing a significant accumulation in G0/G1 cell cycle phase in A172 cells (Supplementary Figure 2). Additionally, a decrease in the cell population in G2/M phase was observed in HIV-1 gp120 clade C treated cells by 22.23% ± 0.62 when compared to control (24.37% ± 0.44) and HIV-1 gp120 clade B treated cells (25.37% ± 0.38). This data is indicative of a cell cycle arrest in G0/G1 in HIV-1 gp120 clade C treated cells (Figure 3A, 3B). Despite that no increase of G2/M cells was observed in HIV-1 gp120 clade B treatment, the >4N population of cells increased in gp120 B (17.23% ± 1.24) when compared to control (11.87% ± 0.90) and HIV-1 gp120 clade C treated cells (12.20% ± 0.10). This may be indicative of polyploid cells, a common effect occurring due to lack of control in mitogenic cell cycle regulators and resulting in accumulation of large amounts of DNA as compared to diploid cells.

To further evaluate the effect of HIV-1 gp120 clade B and C proteins on cell cycle phase transition, Cyclin D1 (*CCND1*) gene expression analysis was performed using qRT-PCR (Figure 3C). Significant increase of CCND1 (3.17 ± 0.38 fold) was identified in HIV-1 gp120 clade B treated cells when compared to control. Similar effect but with smaller magnitude (1.72 ± 0.16 fold) was identified for HIV-1 gp120 clade C treatment. These results suggest that HIV-1 gp120 clade B may alter the cell cycle progression through G1/S transition with *CCND1* expression, possibly contributing to further uncontrolled cell proliferation [39, 40].

CIQBP, a protein with dual function in proliferation and migration was significantly up regulated in HIV-1 gp120 clade B treated cells (Supplementary Table 1). HIV-1 is known to induce chemotaxis/cell migration and activation of resting microglia allowing a productive HIV-1 infection by recruiting and activating these cells at the virus replication sites [41, 42]. To better characterize the effects of HIV-1 gp120 proteins on astrocytoma function, we examined chemotaxis induced in microglia by U87-MG cells treated with HIV-1 gp120 proteins and HIV-1 gp120 proteins alone to test if HIV-1 gp120 proteins alone and/or the cytokines released by the astrocytoma cells may recruit microglia to HIV-1 gp120 sites. Figure 3D demonstrates that HIV-1 gp120 clade B protein alone, increased microglial (HMC3) migration abilities when compared to control. HIV-1 clade gp120 C protein lacked the induction of this migratory effect in HMC3. Additionally, cell migration was performed for HMC3 cells when U87-MG cells at the bottom of the well were treated with HIV-1 gp120 clades B and C proteins (Figure 3E). HIV-1 gp120 clade B treated U87-MG cells showed similar effects as HIV-1 gp120 clade B protein alone, where HMC3 cells showed a higher migration ratio. HIV-1 gp120 clade C treated U87-MG did not show a significant migratory effect. HIV-1 gp120 clade B treated cells showed higher migration abilities when compared to control (1.76 ratio ± 0.30) and HIV-1 gp120 clade C treated cells (0.73 ratio ± 0.07).

To further validate cell migration mechanism induced by HIV-1 gp120, we investigated the involvement of monocyte chemo-attractant protein-1 (*MCP1*) (Figure 3F) and granulocyte colony stimulating factor (*GCSF*) (Figure 3G) in astrocytoma, since these cytokines are key players in microglia’s migration [43–45]. *MCP-1* and *G-CSF* relative gene expression were measured by qRT-PCR in U87-MG after HIV-1 gp120 clades B and C treatment. *MCP-1* chemokine expression was shown to be higher in HIV-1 gp120 clade B treated cells when compared to control (10.23 fold ± 2.16). Non-significant increase of *MCP-1* was observed between control and HIV-1 gp120 clade C or between clades. Moreover, HIV-1 gp120 clade B treated astrocytoma cells showed a significantly higher expression of the G-CSF cytokine when compared to control (5.03 fold ± 0.93), unlike HIV-1 gp120 clade C that did not cause this effect. HIV-1 gp120 clade C treated astrocytoma showed no significant difference of *G-CSF* relative gene expression when compared to control cells. Altogether, these results suggest that not only HIV-1 gp120 clade B treated astrocytoma cells induced microglial migration, but also showed higher expression of key proliferative markers whereas, HIV-1 gp120 clade C showed a G0/G1 cell cycle arrest and lacked the induction of microglial migratory effect.

### HIV-1 gp120 clade C induces cytotoxic effects with the expression of oxidative, inflammatory and key endoplasmic reticulum stress apoptotic markers

Quantitative TMT based proteomics analysis revealed that various biological processes were commonly identified and differentially influenced by HIV-1 gp120 clade B and C treatments. Among the altered biological processes figure proteins involved inimmunological response activation, oxidative and endoplasmic reticulum stress and apoptosis (Table 1 and 2). In order to further validate the involvement of gp120 proteins in the induction of an inflammatory, oxidative and endoplasmic reticulum stress mediated pro-apoptotic response, the role of key markers from these processes were measured together with their cytotoxic effect on the cells.

**Figure 4:**
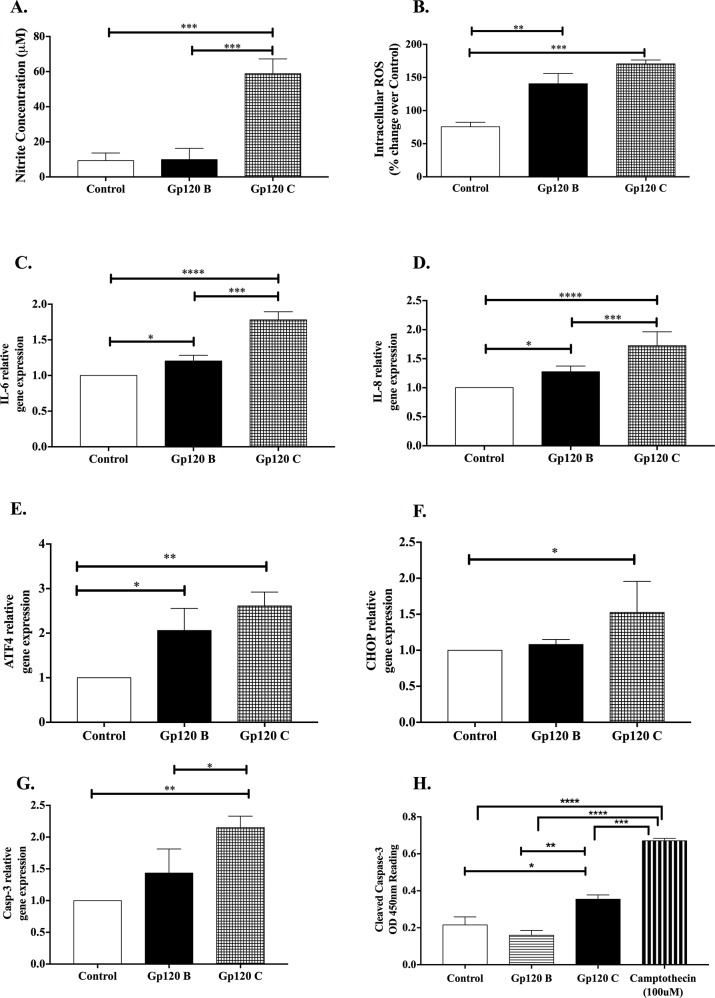
HIV-1 gp120 clade C protein induces cytotoxic effects with the expression of oxidative, inflammatory and key endoplasmic reticulum stress and pro-apoptotic markers **(A)** Nitrite release as an oxidative marker was measured by spectrophotometry with Griess reagent. **(B)** ROS intracellular species production was measured using 2’-7’-dichlorofluorescin diacetate (H_2_DCFDA). Relative gene expression of proinflammatory mediators, IL-6 **(C)** and IL8 **(D)** normalized to GAPDH as housekeeping gene. ER-stress induced apoptotic relative gene expression was measured for ATF4 **(E)**, CHOP **(F)** and CASP3 **(G)**. **(H)** Cleaved caspase 3 protein expression by PathScan Sandwich ELISA. Mean ± SEM and statistical significance was determined using one-way ANOVA, P ≤ 0.05 (N=3).

Oxidative damage induced by HIV-1 gp120 proteins in U87-MG cells was assessed by nitrate release (stable molecule for measuring nitric oxide species, NO) and by the production of reactive oxidative species (ROS) within the cell. A higher nitrite release was observed after gp120 clade C treatment (58.82μM ± 5.95) when compared to gp120 clade B (9.89μM ± 3.71) and control (9.31μM ± 2.48) treatments (Figure 4A). There was no significant difference between control and gp120 clade B treated cells. Moreover, intracellular ROS was induced by HIV-1 gp120 proteins with a higher expression in HIV-1 gp120 clade C treatment when compared to control (170.60% ± 4.10 vs. 75.5% ± 4.00) and gp120 B (170.60% ± 4.10 vs. 140.50% ± 8.95). There was no statistical difference shown between the clades (Figure 4B). These results confirm the deregulation observed in the oxidative stress biological process, together with the detection of several altered proteins identified through the quantitative proteomics TMT approach. Overall, this data further indicates that HIV-1 gp120 clade C induced a higher ROS production than HIV-1 gp120 clade B treated cells.

Moreover, pro-inflammatory mediators such as *IL-6* and *IL-8* relative gene expression were measured in order to validate the quantitative proteomics TMT results demonstrating that gp120 clade B and C proteins differentially altered the immune response by deregulating proteins involved on the regulation of tumor necrosis factor signaling, antiviral mechanism of interferon and activation of NF-kB. It was previously shown that HIV-1 gp120 can induce *IL-8* [46] and *IL-6* expression in astrocytes [47] but a comparative analysis of the differential expression of these neuro-inflammatory markers between clade B and C has never been studied before. Both HIV-1 clades B and C proteins induced the expression of *IL-6* (Figure 4C) and *IL-8* (Figure 4D), however, HIV-1 gp120 clade C treatment showed a higher expression of these two cytokines compared to HIV-1 gp120 clade B treatment (1.78 fold ±0.066 vs.1.20 fold ± 0.04 for *IL-6* and 1.72 fold ± 0.09 vs. 1.27 fold ± 0.04 for *IL-8*, respectively). These results suggest that both HIV-1 gp120 proteins induce a differential inflammatory response in U87-MG cells, while HIV-1 gp120 clade C appears to have a higher inflammatory response as compared to HIV-1 gp120 clade B treated cells.

Furthermore, our results show that HIV-1 gp120 clades B and C proteins induce a protein folding capacity deregulation mediated by the UPR. We speculate that this cytoprotective stress response is activated to cope with ER protein load and pathologic protein aggregation in astrocytoma. It has been previously shown that HIV-1 gp120 protein can induce programmed cell death through the ER stress mechanism [48] but its differential effect between HIV-1 gp120 clades B and C has not yet been investigated. Our results reveal that HIV-1 gp120 clade C treated astrocytoma induced the expression of key ER stress mediated apoptosis markers, such as the activating transcription factor 4 (*ATF4*), caspase 3 (*CASP3)* and CCAAT-enhancer-binding protein homologous protein (*CHOP*), also known as growth arrest and DNA damage-inducible gene 153 (GADD153). Even though the differential expression of ATF4 between HIV-1 gp120 clades B and C was not statistically significant, both HIV-1 gp120 proteins triggered a higher ATF4 gene expression as compared to the control (Figure 4E). Similar results were observed when CHOP was analyzed however, only HIV-1 gp120 clade C treated cells expressed significantly higher gene expression levels (1.53 fold ± 0.31) when compared to control and HIV-1 gp120 clade B (Figure 4F). Another pro-apoptotic marker, tightly linked to chronic ER stress-caspase dependent apoptotic response is *CASP3*. HIV-1 gp120 C led to significant upregulation of gene *CASP3* when compared to control and HIV-1 gp120 B treated cells (1.43 fold ± 0.22 vs. 2.15 fold ± 0.10) (Figure 4G) in astrocytoma. To validate caspase-3 gene expression after gp120 clade B/C treatment, an ELISA based assay was used to detect and quantify the amount of cleaved caspase-3 protein level in U87-MG (Figure 4H) and A172 cells (Supplementary Figure 3). In both cell lines, HIV-1 gp120 clade C treated cells exhibited higher expression of cleaved caspase 3 when compared to control and HIV-1 gp120 clade B treated cells. Camptothecin, was used as positive control of cell death at a concentration of 100μM. Taken together, our findings underline the pro-inflammatory and cytotoxic effects of HIV-1 gp120 clade C in astrocytoma subsequently activating the expression of key chronic ER stress mediated pro-apoptotic markers as compared to HIV-1 gp120 clade B induced cells.

### HIV-1 gp120 clade B induces a protective response triggered by unfolded protein response markers

The quantitative TMT based proteomics analysis (Table 1 and Table 2) revealed that gp120 clade B and C treated astrocytoma deregulate ER-stress/UPR and apoptotic markers. Under conditions when ER stress is chronically prolonged and the protein load on the ER greatly exceeds its fold capacity, cellular dysfunction and cell death often occur. On the other hand, transient acute exposure to ER stress can condition cells for survival.

The survival response was analyzed by qRT-PCR analysis by measuring key UPR/ER Stress markers calcium regulators and intermediates proteins that could determine the final survival or apoptotic response in gp120 clade B treated astrocytoma. The differentially expressed genes included Protein Disulfide Isomerase family A member 5 (*PDIA5*), 78kDa-Glucose Regulating Protein (GRP78 also known as *HSPA5*), eukaryotic initiation factor 2 alpha in its inactive and activate phosphorylated form, calreticulin (*CALR*) and B-cell lymphoma 2 apoptosis regulator (*BCL2*).

**Figure 5:**
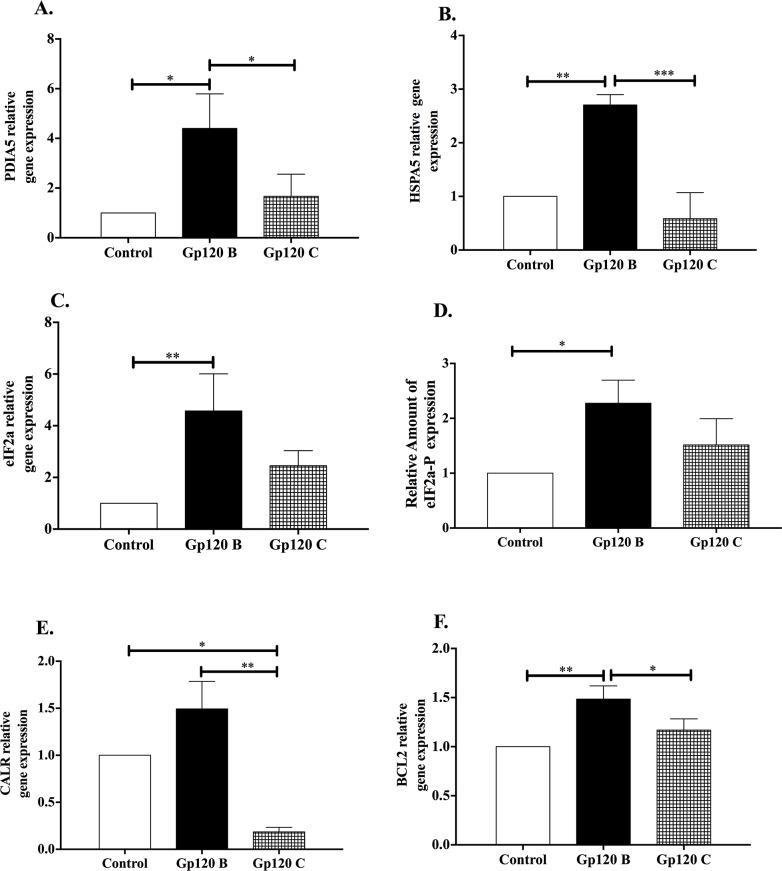
HIV-1 gp120 clade B induces a protective response triggered by Unfolded Protein Response markers UPR key protein markers were measured after HIV-1 gp120 clade B and C astrocytoma treatment. **(A)** PDIA5, **(B)** GRP78, also known as *HSPA5* **(C)** eIF2α relative gene expressions were measured. **(D)** Relative protein amount of the active phosphorylated form of eIF2α (eIF2α-P) was measured through flow cytometry analysis. **(E)** CALR and **(F)** BCL2 relative gene expression measurement. qRT-PCR analysis were normalized with GAPDH as housekeeping gene. Mean ± SEM and statistical significance was determined using one-way ANOVA, P ≤ 0.05 (N=3).

HIV-1 gp120 clade B treated cells showed higher relative gene expression of *PDIA5* (4.40 fold ± 0.80) (Figure 5A) and *HSPA5* (2.70 fold ± 0.11) (Figure 5B) when compared to gp120 C and control. Similar HSPA5 gene expression was observed in A172 after HIV-1 gp120 clades B and C treatments (Supplementary Figure 4). *eIF2*α gene expression was measured in its inactive form (Figure 5C) and the active phosphorylated form (eIF2α-P) protein expression was measured through flow cytometry analysis (Figure 5D). In both cases, HIV-1 gp120 clade B treated astrocytoma cells showed higher gene expression (4.57fold ± 0.83) (Figure 5C) and relative increase in protein amount (2.28 ± 0.24) (Figure 5D) of this ER stress intermediate when compared to control. The data showed no statistical significant differences between HIV-1 gp120 clade C and the control. Moreover, *CALR* and *BCL2* gene expression was measured and showed that HIV-1 gp120 B treated cells induces a higher expression of *CALR* when compared to HIV-1 gp120 clade C and control (1.49 fold ± 0.17) (Figure 5E). Finally, *BCL2*, a protective regulator involved in mitochondrial and ER-stress mediated apoptosis was measured and showed a significantly higher gene expression in HIV-1 gp120 clade B (1.49 fold ± 0.10) (Figure 5F). These results suggest that HIV-1 gp120 clade B protein conferred a protective response while HIV-1 gp120 clade C prompted a more cytotoxic effect in astrocytoma cells.

### Cell surface GRP78 chaperone contributes to HIV-1 gp120 clade B-induced cell survival and proliferative effects in astrocytoma cells

**Figure 6:**
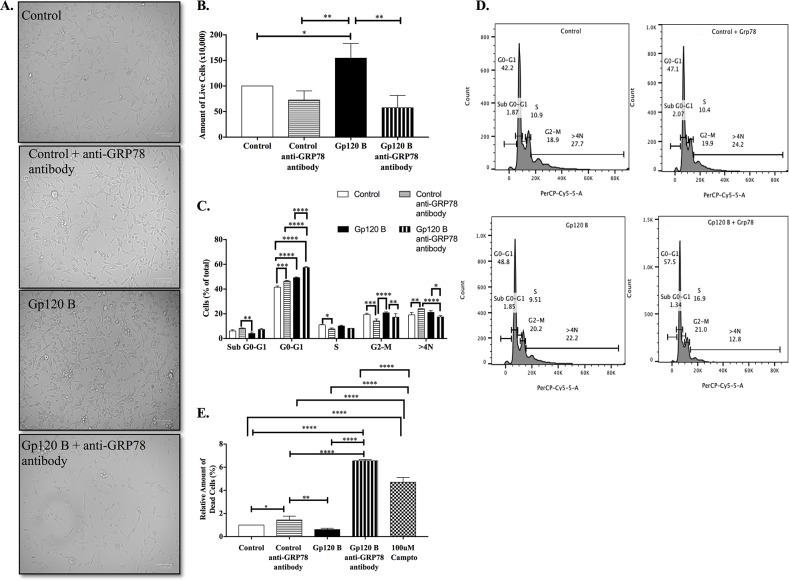
GRP78 contributes to HIV-1 gp120 clade B-induced cell survival and proliferative effects **(A)** Cell density bright-field micrograph at 100um magnification with adjusted +40% brightness for control, +40% contrast for HIV-1 gp120 B and, +20% brightness with +20% contrast for HIV-1 gp120 B with GRP78 mAB. Cell viability was evaluated as a total amount of live cells . **(B)** Cell viability was measured and live cells were counted with use of trypan blue staining. **(C)** Total cell distribution as percentage of all cell cycle stages between treatments. **(D)** Representative histograms from flow cytometry cell cycle analysis with 7-AAD. **(E)** Cell death percentage measured by propidium iodide flow cytometry analysis after HIV-1 gp120 clade B and GRP78mAB treatments. Mean ± SEM and statistical significance was determined using one-way ANOVA, P ≤ 0.05 (N=3).

GRP78 has been previously shown to be involved in the regulation of cell survival, proliferative effects and immune-recognition cells [31, 33, 49]. HIV-1 gp120 clade B treated astrocytoma cells uniquely expressed GRP78 (*HSPA5*) gene unlike gp120 clade C treatment, prompting a more protective and proliferative response in the case of HIV-1 gp120 clade B. Subsequently, we investigated the effect of the cell surface GRP78 chaperone and its contribution to induce a cellular proliferative response in HIV-1 gp120 clade B and C treated cells. To assess this, astrocytoma cells were exposed to GRP78 monoclonal antibody followed by HIV-1 gp120 clade B treatment. Trypan blue was used to determine the amount of cell viability after HIV-1 gp120 clade B treatment in astrocytoma. Cells treated with HIV-1 gp120 clade B showed an increased amount of live cells when compared to control alone (154.50 ± 16.55) and to control with GRP78 mAB (72.50 ± 10.33). Moreover, HIV-1 gp120 clade B depicted a higher amount of live cells when compared to HIV-1 gp120 clade B with GRP78 mAB (154.50 ± 16.55 vs. 57.57± 13.88). Cell treatment micrographs highlight cell density as revealed in Figures 6A and 6B.

Cell cycle effect induced by HIV-1 gp120 clade B protein was evaluated by flow cytometry in astrocytoma U87-MG cells. We observed a lower sub G0-G1 population in HIV-1 gp120 clade B treatment. This is commonly associated with apoptosis when compared to control with GRP78 mAB (Figure 6C). Furthermore, HIV-1 gp120 clade B treated cells with GRP78 mAB had a higher accumulation of cells in G0/G1 cell cycle phase when compared to control (57.45% ± 0.45 vs. 41.50% ± 0.70), control with GRP78 mAB (46.43 % ± 0.33), and HIV-1 gp120 clade B alone (49.20% ± 0.40). A decrease in cell population in S phase in the control with GRP78 mAB group was observed when compared to control alone (7.80% ± 0.55). A decrease in G2/M cell population was also observed between control and control with GRP78 mAB and between HIV-1 gp120 clade B when compared with HIV-1 gp120 clade B with GRP78 mAb group. The control with GRP78 mAb treated cells revealed an increase in >4N population when compared to control and HIV-1 gp120 clade B with GRP78 mAb group. This possibly is indicative of polyploid cells. Figure 6D shows the representative histograms from flow cytometry cell cycle analysis. These results indicate that neutralizing GRP78 cell surface expression with a GRP78 mAB seems to prompt a cell cycle arrest in control cells with GRP78 mAB and in HIV-1 gp120 B cells with GRP78, with higher amount of accumulated cells in the latter.

To further validate the neutralization outcome of cell surface protein GRP78 with a mAB on cell viability and proliferation in HIV-1 gp120 clade B treated cells, the amount of cell death was subsequently measured by flow cytometry analysis using propidium iodide. Figure 6E depicts that the relative amount of dead cells increased when GRP78 mAB was added to treatments as observed between control and control with GRP78 mAB (1.00% ± 0.00 vs 1.45% ± 0.16) and between HIV-1 gp120 clade B and HIV-1 gp120 clade B plus GRP78 mAB groups (0.62% ± 0.06 vs 6.56% ± 0.09). Cells treated with the apoptosis inducer camptothecin at 100uM, were used as a positive control. Our results imply that GRP78 plays a pivotal role in the HIV-1 gp120 clade B-induced effect on astrocytoma cell survival and proliferation and that neutralizing this protein function may revoke this protective effect into a more cytotoxic one.

## DISCUSSION

The diversity in HIV-1 clades may lead to differential expression of HAND and may cause differences in the progression of disease pathogenesis, making it a major obstacle for developing alternative treatments. HIV-Associated Neurocognitive Disorder (HAND) neuropathogenesis markers have been shown to be more common in HIV-1 clade B than clade C [50], however the mechanism behind this differential response remains to be elucidated.

The present study sought to determine whether HIV-1 gp120 clade B and C proteins differentially induce the impairment of multiple signaling pathways implicated in proliferation, ER stress/UPR, cell survival and NeuroAIDS in human astrocytoma. After 24 hours of HIV-1 gp120 clade B and C treatment, a significant deregulation in cell density between clades was observed. HIV-1 gp120 clade B conferred increased cell viability when compared to control cells, while HIV-1 gp120 clade C induced an increase in cell death. These findings suggest that HIV-1 gp120 clade B and C proteins may have differential effects in cell cytotoxicity. Previous studies demonstrated that HIV protein Tat induced differential cytotoxic response among clades [51] and to our knowledge this is the first report of a quantitative proteomics dataset in astrocytoma which examine HIV gp120 clade B and C differential effects. TMT isobaric labeling quantitative proteomic approach has been successfully applied to this study as a powerful tool for accurate quantification and identification of novel protein signatures with differential cytotoxic, survival and pro-apoptotic responses. The differentially expressed proteins identified validate the study hypothesis on the differential cytotoxic response among HIV-1 gp120 clades B and C.

Our findings establish that the unfolded protein response (UPR) plays as an activator of pro-survival pathways in HIV gp120 clade B induced astrocytoma. UPR is a cytoprotective response to acute or chronic ER stress, activated in response to accumulation of misfolded proteins in the lumen of the endoplasmic reticulum. Downstream signaling dictate opposing cell fates (survival or death) during ER stress depending on the cellular coping mechanisms activated to minimize accumulation and aggregation of misfolded proteins, by increasing the capacity of the ER machinery for folding and degradation. The results obtained suggest that PERK acts as the main receptor activated by both HIV-1 gp120 B and C clades, leading to the activation of PERK’s either anti or pro-apoptotic downstream signaling. PERK-eIF2α-P pathway has a dual function in cell survival and apoptosis by regulating PTEN and PI3K-Akt signaling in different cell models [52, 54] and by causing a translational attenuation, leading to a cell cycle arrest [55, 56]. These studies support our findings highlighting a cell cycle arrest in G0/G1 phase, and the upregulation of 14-3-3 zeta/delta and theta proteins, associated with DNA damage checkpoint in HIV-1 gp120 clade C treated cells. Even though HIV-1 gp120 clade C prompted a pro-apoptotic response, the cell cycle arrest results (Figure 3B) suggest that the cell is struggling to survive by triggering the upregulation of proteosomal subunits α/δ proteins, associated with ERAD. This activation demonstrates an unsuccessful coping mechanism to prevent cell death. In contrast, HIV-1 gp120 clade B exerted an increased expression in CDC6 and CCND1; positive regulators of cell cycle G1/S phase transition markers employed as a proliferative response and a survival outcome downstream mechanism, involving anti-apoptotic markers such BCL-2 (Figure 5F).

Additionally, the activation of eIF2α by its phosphorylation led to the expression of ATF4, which was highly expressed in HIV-1 gp120 clade B and C treatments, with higher expression in HIV-1 gp120 clade C treated cells. ATF4 is known to translocate to the nucleus and transcribe chaperones such as GRP78, calreticulin and PDI [57]. The production of such proteins result in a protective survival response by re-establishing a correct folding of proteins [58]. Although ATF4 could potentially activate a survival mechanism under acute ER stress, it has also the potential to promote a chronic ER stress mediated via CHOP apoptotic signal [57] through the PERK-eIF2-ATF4-CHOP–axis.

The activation of CHOP consequently leads to a caspase cell death signaling, including the downstream apoptotic marker caspase-3 [59, 60]. HIV-1 gp120 clade B induced the upregulation of GRP78, a master regulator of UPR exerting a cell survival response, while HIV-1 gp120 clade C prompted a pro-apoptotic response through the increased expression of CHOP, reactive oxidative species and caspase-3. Interestingly, the presence of such toxic environment produced an inflammatory response, by inducing the overexpression of IL-6 and IL-8, contributing to neuropathogenesis, confirmed by previous studies [61]. Conversely, HIV-1 gp120 clade B induced a higher expression of G-CSF, known to induce neuroprotection and chemotaxis [62, 63]. Chemotaxis in HIV infection is not only involved in antigen presentation but also facilitates viral transmission [64]. The chemotaxis results from this study demonstrated that HIV-1 gp120 clade B protein conferred a microglial migratory effect that was not only induced by the protein itself, but also by the release of inflammatory mediators.

Furthermore, HIV-1 gp120 clade B treatment induced the expression of a negative regulator marker involved in neuron death by oxidative damage, GNB2L1, also known as RACK1 (receptor for activated C-kinase 1). This anchoring protein that shuttles activated PKC to cellular membranes, has a key function in PKC-mediated signal transduction pathways that may underlie muscarinic regulation impairment due to β-amyloid [65]. Significant loss of RACK1 has been associated with dementia [65]. Its presence in HIV-1 gp120 clade B treated astrocytoma, suggests that it has a protecting effect in the cell by decreasing the oxidative damage and negatively regulating the formation of amyloid fibers. On the other hand, HIV-1 gp120 clade C induced the expression of amyloid fiber formation related proteins.

Moreover, following HIV-1 gp120 clade B treatment, the expression level of the ER-stress suppressor of apoptosis Bcl-2 protein was significantly increased in astrocytoma. Besides Bcl-2 known role in regulating intrinsic mitochondrial apoptosis, this anti-apoptotic protein is known to be activated by ER stress response to induce calcium release to the cytosol [66]. Bcl-2 and calreticulin, were both also induced by HIV-1 gp120 clade B as revealed by the quantitative proteomics and qRT-PCR results (Table 1 and Figure 5E and 5F). Bcl-2 combined with calreticulin blocks or decreases calcium concentration from the ER and modulates its entrance to the mitochondria [66, 67], contributing to mitochondrial calcium homeostasis. This essential process determines an increase in cytochrome C release possibly causing ROS and/or cell death [66]. Additionally, Bcl-2 can act as a blocker for CHOP-induced apoptosis leading to cell survival [68]. An overexpression of this protein may negatively regulate CHOP expression and consequently inhibits CHOP-apoptotic effect induced by the ER [69]. CHOP may also repress Bcl-2 protective role expression leading to cell death [70, 71]. The unique expression of CHOP in HIV-1 gp120 clade C and Bcl-2 in HIV-1 gp120 clade B reaffirm that HIV-1 gp120 proteins exert a clade dependent specific response in cell death through UPR activation in U87 astrocytoma cells.

As a key player of UPR, GRP78 triggers survival-signaling cascade by detaching from the UPR receptors. Furthermore, it has been associated with numerous functions besides the ER stress response and has been proved to be up-regulated in HIV-infected and neurocognitively impaired individuals [72], suggesting GRP78’s role in HIV-induced neurodegeneration. The results from this current study indicate that HIV-1 gp120 clade B induced GRP78 specific upregulation, confirming the protective response obtained in astrocytoma cells. Additionally, the blocking of extracellular GRP78 in HIV-1 gp120 clade B validates its proliferative and protective effect by reverting it.

**Figure 7:**
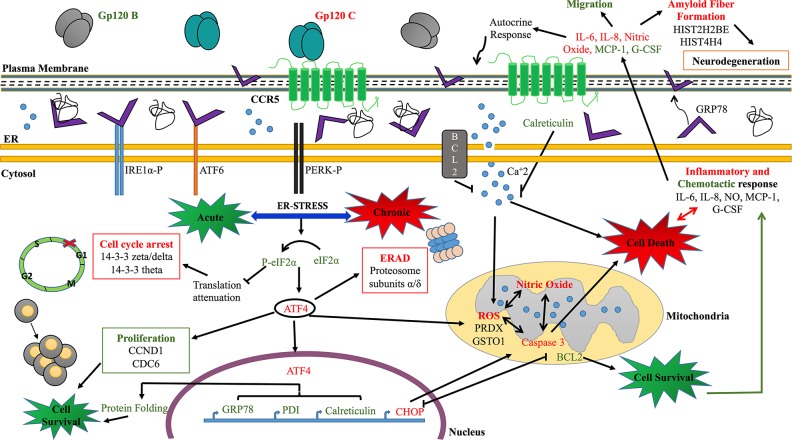
Proposed model highlighting differential acute and chronic ER stress leading to pro-apoptotic or cell survival responses in HIV-1 gp120 clade B and C treated astrocytoma The model highlights the protective role induced by HIV-1 gp120 clade B in a proliferative, migratory and acute ER-stress type of response buffered by the UPR survival chaperone GRP78. Insufficient folding or degradation capacity in HIV-1 gp120 C treated cells promoted cell death induced by a chronic, severe ER stress response mediated by G0/G1 cell cycle arrest, oxidative, inflammation and autophagy response leading to apoptosis. Examples of proteins identified by quantitative proteomics isobaric TMT labeling, are highlighted with their corresponding biological function. HIV-1 gp120 clade B and C induced altered biological processes and differential expression of key protein signatures represented in green and red respectively.

This study is the first integrated Tandem Mass Tag (TMT) quantitative mass spectrometry-based analysis on the differential effect of HIV-1 gp120 clade B/C in astrocytoma. Our dataset provide insight into neuropathogenesis of HIV-1 clade B and C differentiation. Our proposed model highlights that HIV-1 gp120 clade B and C prompted the impairment of multiple signaling pathways, including ER stress, inflammation, migration, proliferation, cell cycle arrest, pro-apoptotic and survival mechanisms that may culminate in neurodegeneration (Figure 7). HIV-1 gp120 proteins have demonstrated to induce a clade specific UPR mediated response. We conclude that HIV-1 gp120 B induced a more protective and proliferative role, while HIV-1 gp120 clade C induced a cytotoxic effect. The proliferative and protective role of HIV-1 gp120 clade B can be bestowed to a survival mechanism triggered by GRP78. Taken together, our results suggest that HIV-1 gp120 clade B had a more neuropathogenic role due to the continual proliferation and migration of cells that may further exacerbate HIV-1 viral replication. We believe that this study is a significant contribution to the fields of NeuroAIDS knowledge in HIV-1 clade differentiation that could potentially reveal therapeutic targets and mechanistic strategies against HIV-1 gp120 clade B and C.

## MATERIALS AND METHODS

### HIV-1 gp120 clade B and C protein treatment

U87-MG astrocytoma cells, which have been frequently used to analyze the effect of HIV-1 gp120 clade B and Cproteins and neuropathogenesis were used for this study [73, 74]. Human astrocytoma cell lines U87-MG and A172 (American Type Culture Collection, ATCC, Manassas, VA), used in the supplementary data as a further result validation, were both cultured in Eagle’s Minimum Essential Medium (EMEM; ATCC, Manassas, VA), supplemented with 10% heat inactivated fetal bovine serum (FBS; ATCC, Manassas, VA) and penicillin/streptomycin (100 U/mL and 100 μg/mL; Invitrogen). Cells were grown to 80-90% confluence and then plated in 6 well tissue culture plates at densities of 1×10^6^ cells ml/well. HIV-1 gp120 clade B (Bal) and gp120 clade C (96ZM651) proteins were obtained from NIH AIDS Research and Reference Reagent. These recombinant gp120 clade B protein and gp120 clade C protein were >95% and 90% purified respectively. A solution of phosphate buffered saline (PBS) was subsequently diluted in media to the required concentrations of HIV-1 gp120. For all experiments, cells treated with vehicle alone (media alone) were used as the untreated control. U87-MG and A172 cells were separately treated with HIV-1 gp120 clade B and C at 200ng/ml for 24 hours. Cells were then maintained in a cell culture incubator at 37°C in a humidified atmosphere with 5% CO_2_ and cell density images were taken at 100um in brightfield with a ZOE Fluorescent Cell Imager (Biorad, Hercules, CA).

### HIV-1 clade B gp120 protein treatment with anti GRP78

Approximately 50,000 cells/well of U87-MG were cultured in a 24-well plate (Sigma-Aldrich, Saint Louis, MO) in EMEM (ATCC, Manassas, VA), supplemented with 10% heat inactivated FBS (ATCC, Manassas, VA) and penicillin/streptomycin (100 U/mL and 100 μg/mL; Invitrogen). Cells were then treated in presence or absence of 2μg/ml monoclonal rabbit anti GRP78 antibody (mAB) (Cell Signaling, Danvers, MA) for 30 minutes. After 30 minutes, cells were then treated in presence or absence of 200ng/ml of gp120 clade B (Bal) protein treatment followed by 24 hours incubation. For all experiments involving GRP78, cells treated with vehicle alone (media alone) were used as the untreated control. Cells were maintained in a cell culture incubator at 37°C in a humidified atmosphere with 5% CO_2_. Cell density images after cells treated with HIV-1 gp120 clade B and anti GRP78 antibody were taken at 100um in brightfield with a ZOE Fluorescent Cell Imager (Biorad, Hercules, CA).

### Trypan blue viability assay

U87-MG and A172 cells were trypsinized (0.25%Trypsin/0.53mM EDTA, American Type Culture Collection, ATCC, Manassas, VA) and stained with Trypan Blueand the total number of live and dead cells were determined by cell counting. Viability measurement was performed with 0.4% trypan blue in PBS solution (Hyclone, Pittsburgh, PA) then was mixed with each of the cell aliquots, using 75 μL of stain to 25 μL of cell suspension. The suspension was loaded into a hemocytometer and followed by counting the cells. Cells that stained blue were scored as nonviable.

### Isobaric labeling tandem mass tag (TMT) quantitative proteomic approach

Tandem Mass Tag (TMT) isobaric labeling quantitative proteomic analysis was performed as previously described [75]. TMT’s with varying molecular weights (126–131 Da) (Thermo Scientific, Waltham, MA) were used as isobaric labels to determine differential protein expression between U87-MG cells treated with HIV-1 gp120 clade B and C proteins at 200ng/ml for 24 hours and U87-MG astrocytoma cells without protein treatment as a control. According to manufacturer’s protocols, the six digested samples were individually labeled with TMT6 reagents as follows. Two control (non-treated) samples were labeled with TMT-126 and TMT-127. Two gp120 B treated astrocytoma samples were labeled with TMT-128 and TMT-129. Two gp120 C treated astrocytoma samples were labeled with TMT-130 and TMT-131. The labeled peptide mixtures were combined in equal ratios.

### Database search and TMT quantification

The protein search algorithm SEQUEST was used to identify unique protein peptides using the Proteome Discoverer data processing software (version 1.2, Thermo Fisher Scientific, Waltham, MA). The ratios of TMT reporter ion abundances in MS/MS spectra generated by HCD from raw data sets were used For TMT quantification. Data was normalized by the relative abundance of the each independent treatment versus control. TMT signals were used to determine protein expression ratios between cells treated with HIV-1 gp120 B or gp120 C. Each HIV-1 gp120 protein condition was normalized to control by using the relative abundance. After the relative abundance was obtained from each treatment individually, the fold change was calculated by using the relative protein abundance of HIV-1 gp120 B and C treated astrocytoma.

### Bioinformatics analysis of data

Bioinformatics analysis was performed using the Gene Set Enrichment Analysis approach (GSEA). The method derives its power by focusing on gene sets that share common biological function or regulation as previously described [76].

### Cell cycle analysis

1×10^6^ U87-MG and A172 cells were used for cell cycle analysis after HIV-1 gp120 clade B and C treatment and approximately 150,000 cells were used for gp120 B with anti GRP78 antibody experiments in U87-MG. Cells were washed with phosphate-buffered saline (PBS) and fixed gently (drop by drop) in 70% ethanol for 30 minutes at 4°C. The cells were then resuspended in PBS containing 1μg/ml 7-aminoactinomycin D (7-AAD; Bio-Rad, Hercules, CA, USA) 0.2 mg/ml RNase A (Sigma-Aldrich, St. Louis, MO, USA), incubated for 30 min at 37°C in the dark, and then analyzed with a flow cytometer FACSCanto II (Becton Dickinson, San Jose, CA, USA). DNA content data to determine the percentage of cells in G_0_/G_1_, S and G_2_/M phases was established using FlowJo data analysis software v. 10 (Ashland, OR).

### Migration assay

Migration assays were performed for HMC3 microglial cells using Fluoroblok inserts (8 μM pore size, VWR Scientific, Radnor, PA). 50,000 U87-MG cells were seeded on the bottom of 24 well plate (Sigma-Aldrich, Saint Louis, MO) and treated with gp120 proteins for 24 hours. Serum-starved microglia HMC3 cells were placed on the insert membrane at a cell density of 20,000 cells with FBS-free medium. 10% FBS or 0% FBS in the lower chamber were used for the positive and negative control, respectively. After 5 hours, cells were fixed with 70% ethanol and stained for 15 mins with 3mM propidium iodide. Cells that migrated to the lower chamber were determined by counting the number of fluorescent cells.

### Quantitative real-time PCR analysis

Expression of IL-6, IL-8, MCP-1, G-CSF, GRP78, PDI, Calreticulin, eIF2α, CHOP, Bcl-2, Caspase-3, ATF-4 and Cyclin D1 was analyzed using qRT-PCR. RNA was extracted from cell pellets using TRIzol reagent (Life Technologies, Carlsbad, CA) following the manufacturer’s protocol. RNA quality and concentration was quantified spectrophotometrically with the NanoDrop 1000 spectrophotometer (Thermo Scientific, Waltham, MA). cDNA was reverse-transcribed from 1 μg of total RNA using the iScript cDNA synthesis kit (Bio-Rad, Hercules, CA). Taqman gene expression assays (Applied Biosystems, Foster City, CA) was used for the following genes: Calreticulin (*CALR*, assay ID Hs00189032_m1), 78-kDa glucose-regulated protein (GRP78, also known as *HSPA5*, assay ID Hs00607129_gH), eukaryotic initiation factor 2 alpha (*eIF2α*, assay ID Hs00260491_m1), B cell lymphoma-2 (*BCL2*, Hs00608023_m1), CCAAT/Enhancer-Binding Protein Homologous Protein (*CHOP*, Hs00358796_g1DDIT3) and protein disulfide isomerase A4 (*P4HB*, Assay ID Hs00168586_m1). All data were controlled for the quantity of RNA input by glyceraldehyde 3-phosphate dehydrogenase (*GAPDH*, assay ID Hs99999905_m1) serving as the endogenous control and for normalization (results were normalized with the control column). Reactions were run on a CFX96 Real Time System C1000 Touch Thermal Cycler (Bio-Rad Laboratories, Hercules, CA) using the following program:10 minutes at 95°C, 39 cycles of 15 seconds at 95°C, and 1 minute at 60°C for annealing temperature. Taqman assay was used to measure GRP78 gene expression by qRT-PCR in A172 cells similarly to the protocol described for U87 cells.

SYBR green qRT-PCR gene expression assays were performed for the following genes: Interleukin-6 (*IL-6*), Interleukin-8 (*IL-8*), Monocyte chemoattractant protein-1 (*MCP-1*), Granulocyte colony stimulating factor (*G-CSF*), Caspase-3 (*CASP3*), Activating transcription factor-4 (*ATF4*) and Cyclin D1 (*CCND1*) using 50 nM Sigma-Aldrich (Saint Louis, MO) primers. Amplification was carried out in a Bio-Rad CFX96 Touch real-time PCR detection system (Bio-Rad, Hercules, CA) using the following program: 10 minutes at 95°C, 50 cycles of 15 seconds at 95°C, and 1 minute at the gene-specific annealing temperature. The gene-specific primers were: *IL-6* forward, 5’-CACCTCAGATTGTTGTTGTT-3’; *IL-6* reverse, 5’-AATAGTGTCCTAACGCTCATA-3’; *IL-8* forward 5’-AAGGAACCATCTCACTGTGTGTAAAC-3’; *IL-8* reverse, 5’-GGAAGGCTGCCAAGAG-3’; *MCP-1* forward 5’-ATAGCAGCCACCTTCATT-3’; *MCP-1* reverse 5’-CTGAGTCACCTTATCTTGGA-3’; *G-CSF* forward 5’-GGGAGGTAGATAGGTAAA-3’; *G-CSF* reverse 5’-ATATTAAACAGGGATTTCTTG-3’; *CASP-3* forward 5’-AAATGAATGGGCTGAGCTGC-3’; *CASP-3* reverse 5’-GCGTATGGAGAAATGGGCTG-3’; *ATF-4* forward 5’-CCTTCTTACAACCTCTTC-3’; *ATF-4* reverse 5’-CATACAGATGCCACTATC-3’; *CCND1* forward 5’-TTCTCCTTGTTGTTGGTT-3’; *CCND1* reverse 5’-TCCTCTATCATCTGTAGCA-3’. The gene expression level was defined as the threshold cycle number (CT). Mean fold changes in expression of the target genes were calculated using the comparative CT method (RU, 2–ΔΔCt). All data were controlled for the quantity of RNA input by *GAPDH* forward 5’-CTGGGCTACACTGAGCACC-3’; *GAPDH* reverse 5’-AAGTGGTCGTTGAGGGCAATG-3’ serving as the endogenous control and for normalization (results were normalized with the control column).

### Phosphorylated-eukaryotic initiation factor 2-alpha (eIF2α -p) expression analysis

The expression of eIF2α-p was analyzed by flow cytometry using approximately 1×10^6^ U87-MG cells with BD FACsCanto II flow cytometer by indirect staining with 1:100 rabbit anti- eIF2α-p (Ser51) antibody (Cell Signaling, Danvers, MA) and a donkey anti-rabbit secondary antibody conjugated with Alexa Fluor 594 (1:2000; Abcam, Cambridge, MA). Data analysis was performed using FlowJo data analysis software v. 10 (Ashland, OR) and results were normalized with the control column.

### Reactive oxidative species assay

After 24hr treatment with HIV-1 gp120 clades B and C, intracellular generation of H_2_O_2_ oxidative species was assessed using the fluorescence indicator 2’-7’-dichlorofluorescin diacetate (H_2_DCFDA, Molecular Probes, Eugene, OR). U87-MG were incubated in a 96-well microplate and loaded for 1 hour with 20μM H_2_DCFDA in PBS. Then, the buffer was removed and the cells were returned to EMEM culture growth medium and incubated at 37˚C, as a short recovery step to render the dye response to oxidation. Fluorescence was measured with a microplate reader Infinite 200M Pro (Tecan, Morrisville, NC) at Excitation 485nm and Emission 520nm.

### Nitrite measurement

After treatment of astrocytoma HIV-1 gp120 clades B and C, nitrite (NO_2_^−^) release, the stable product of nitric oxide was measured in culture medium using the Griess reagent (Promega, Madison, WI) following manufacturer protocol. Briefly, Griess reagent (freshly made before use) consisting of equal volumes of 0.1% naphthylenediamine dihydrochloride in distilled H_2_O and 1% sulfanilamide and 6% H_3_PO_4_ in distilled H_2_O, was added in equal volume to astrocytoma culture supernatants. After 10 minutes incubation at room temperature (away from light), the samples absorbance were read and analyzed using SoftMax Pro software (Molecular Devices, Sunnyvale, CA) coupled to a VersaMax Tunable microplate reader (Molecular Devices, Sunnyvale, CA) detecting at 520nm excitation 550nm emission. NO_2_^−^ concentration was determined from a standard curve generated with a series of concentrations of sodium nitrite (NaNO_2_, from 0-100μM).

### Cell apoptosis by propidium iodide staining

Cell death validation was performed using propidium iodide (solution 1mg/ml in water, Sigma-Aldrich, Saint Louis, MO) in U87-MG and A172 cells after HIV-1 gp120 clade B/C treatment and in U87-MG treated with HIV-1 gp120 clade B plus anti GRP78 antibody treatment Approximately 300,000 U87-MG and A172 cells were used and washed with 3% FBS/PBS followed by an incubation of 10min in the dark with 0.01μg of propidium iodide in 500μl 3% FBS-PBS and then analyzed with a flow cytometer FACSCanto II (Becton Dickinson, San Jose, CA, USA). Data analysis was performed using FlowJo data analysis software v. 10 (Ashland, OR). Positive control for cellular apoptosis was achieved with 100μM Camptothecin diluted in dimethyl sulfoxide (DMSO, Sigma-Aldrich, Saint Louis, MO) for 24 hours. Results were normalized with the control column.

### Cleaved caspase-3 sandwich ELISA

Activation of Caspase-3 by cleavage was performed as a validation for cell death and caspase-3 gene expression. Quantification of cleaved caspase-3 was performed for U87-MG and A172 cells using the PathScan Cleaved Caspase-3 (Asp175) sandwich ELISA kit (Cell Signaling, Danvers, MA). Briefly, after gp120 B/C treatment, the cells were washed with cold PBS and lysed with RIPA buffer (1.5 M Tris pH 8.8, 1.75 g NaCl, 2 mL of 10% sodium 255 dodecyl sulfate, 2 mL Triton X-100; all reagents from Thermo Fisher Scientific, Waltham, MA). Followed by sonication, samples were centrifuged for 10min (x14, 000 rpm) at 4°C. Samples were incubated in the plate for 2 hours at 37°C and washed with 1X wash buffer. Plate was incubated with detection antibody at 37°C for 1 hour, followed by HRP-linked secondary antibody incubation for 30 minutes at 37°C and finalized with TMB substrate incibation for 10 minutes at 37°C. Absorbance was measured and analyzed using SoftMax Pro software (Molecular Devices, Sunnyvale, CA) coupled to a VersaMax Tunable microplate reader (Molecular Devices, Sunnyvale, CA) detecting at 450nm. Positive control for cellular apoptosis was achieved with 100μM Camptothecin diluted in dimethyl sulfoxide (DMSO, Sigma-Aldrich, Saint Louis, MO) for 24 hours.

### Statistical analysis

All experiments were performed in biological triplicates. Statistical analysis was performed through one-way or two-way ANOVA analysis followed by Tukey test, as appropriate, using GraphPad Prism version 7.01 statistical software (GraphPad Software, La Jolla, CA). P-values ≤ 0.05 were considered statistically significant, and all data are expressed as mean ± standard error of mean (mean ± SEM).

## SUPPLEMENTARY MATERIALS FIGURES AND TABLES







## Abbreviations

7-AAD: 7-aminoactinomycin D
AIDS: acquired immunodeficiency syndrome
ATF4: activating transcription factor 4
ATF6: activating transcription factor 6
BCL2: B cell lymphoma-2
BBB: blood brain barrier
CALR: calreticulin
CASP3: caspase 3
CCND1: cyclin D1
CHOP: CCAAT-enhancer-binding protein homolo-gous protein
CNS: central nervous system
CT: threshold cycle number
EDTA: ethylenediamine tetraacetic acid
eIF2α: eukaryotic initiation factor 2 alpha
eIF2α-P: phosphorylated eukaryotic initiation factor 2-alpha
EMEM: eagle’s minimum essential medium
ER: endoplasmic reticulum
ERAD: endoplasmic reticulum associated protein degradation process
FBS: fetal bovine serum
FDR: false discovery rate
GAPDH: glyceraldehyde-3-phosphate dehydro-genase
G-CSF: granulocyte colony stimulating factor
GRP78, HSPA5: glucose regulating protein 78 KDa
GSEA: gene set enrichment analysis
HAD: HIV-associated dementia
HAND: HIV-associated neurocognitive disorders
HAART: highly active antiretroviral therapy
HCD: high-energy collision dissociation
HIV-1: human immunodeficiency virus type 1
HPLC: high-performance liquid chromatography
IL-6: interleukin-6
IL-8: interleukin-8
IRE1α: inositol requiring enzyme-1- alpha
LC-MS/MS: liquid chromatography- mass spectrometry
mAB: monoclonal antibody
MCP-1: monocyte chemoattractant protein-1
MS: mass spectrometer
NO: nitric oxide
PBS: phosphate buffered saline
PDIA5: protein disulfide isomerase A5
PERK: protein RNA kinase-like endoplasmic reticulum kinase
qRT-PCR: quantitative reverse transcriptase polymerase chain reaction
RNS: reactive nitrogen species
ROS: reactive oxidative species
SCX: strong cation-exchange
SPE: solid-phase extraction
TFA: trifluoroacetic acid
TMT: tandem mass tag
UPR: unfolded protein response
