# Supplementary material for: HIV-1 Gp120 clade B/C induces a GRP78 driven cytoprotective mechanism in astrocytoma
Source: Oncotarget. 2017 Jul 22;8(40):68415–38. doi: 10.18632/oncotarget.19474 (PMC5620267; doi:10.18632/oncotarget.19474)
Supplement: Supplementary file 1 [file oncotarget-08-68415-s001.pdf]

HIV-1 Gp120 clade B/C induces a GRP78 driven cytoprotective mechanism in astrocytoma

SUPPLEMENTARY MATERIALS

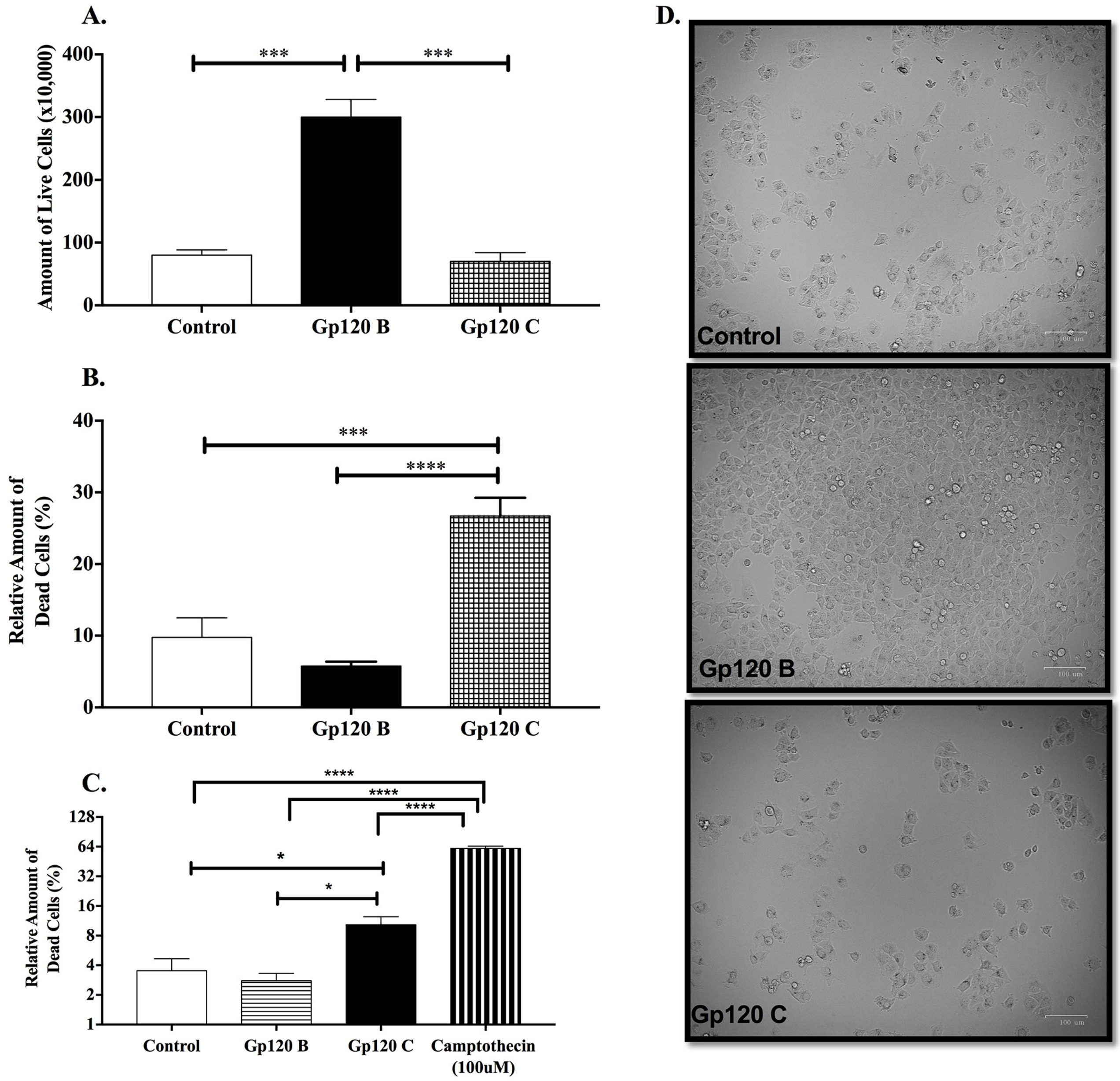

Supplementary Figure 1: HIV-1 gp120 clade B and C proteins induced a differential interclade effect in cell viability and cell death in A172 cells. Experiments were performed for control A172 vehicle (medium only) and cells treated with HIV-1 gp120 clades B and C for 24 hours. Live and dead cells were counted with use of trypan blue staining. **(A)** Cell viability was evaluated as a total amount of live cells. **(B)** Relative amount of dead cells was evaluated as percent equivalent to the total amount of cells. **(C)** Cell death percentage was measured by propidium iodide flow cytometry analysis after HIV-1 gp120 clade B and C treatments. **(D)** Cell density bright-field micrographs at 100um magnification after HIV-1 gp120 clade B and C treatment. Mean ± SEM and statistical significance was determined using one-way ANOVA, $P \leq 0.05$ (N=3).

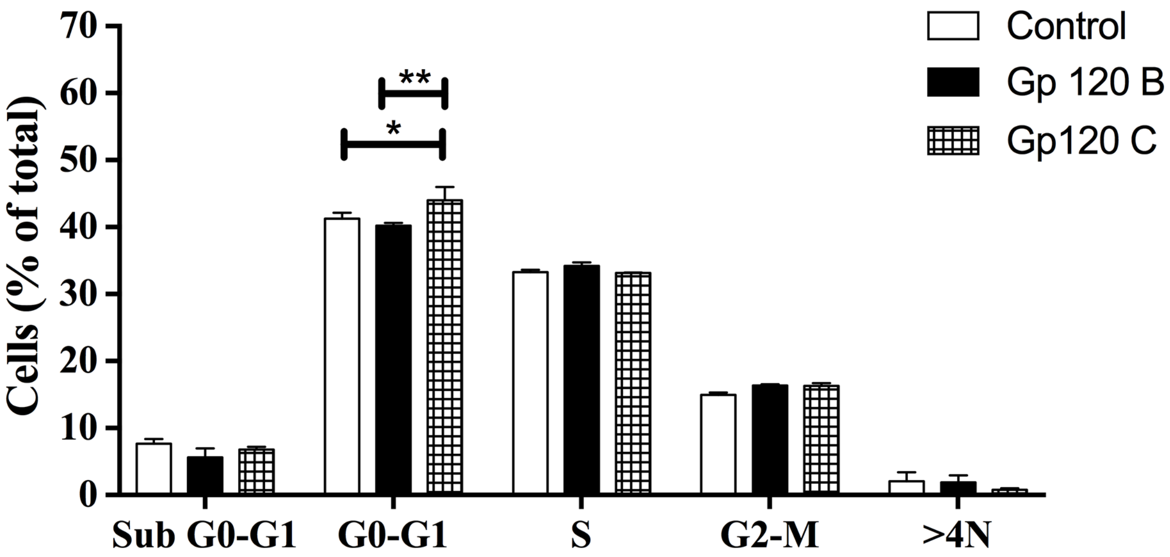

Supplementary Figure 2: HIV-1 clade C gp120 protein induces G0/G1 cell cycle arrest in A172 cells. Flow cytometry analysis with use of 7-aminoactinomycin D staining and PerCP-Cy5-5-A filter set was used for the identification of cell cycle. The percentage of cells in G0/G1, S, and G2/M phases was identified based on DNA content. The graph represents the total distribution of cells at different phases of the cell cycle. Amount of cells at each phase is shown as a percentage of the total amount of cells. Mean ± SEM and statistical significance was determined using two-way ANOVA, $P \leq 0.05$ (N=3).

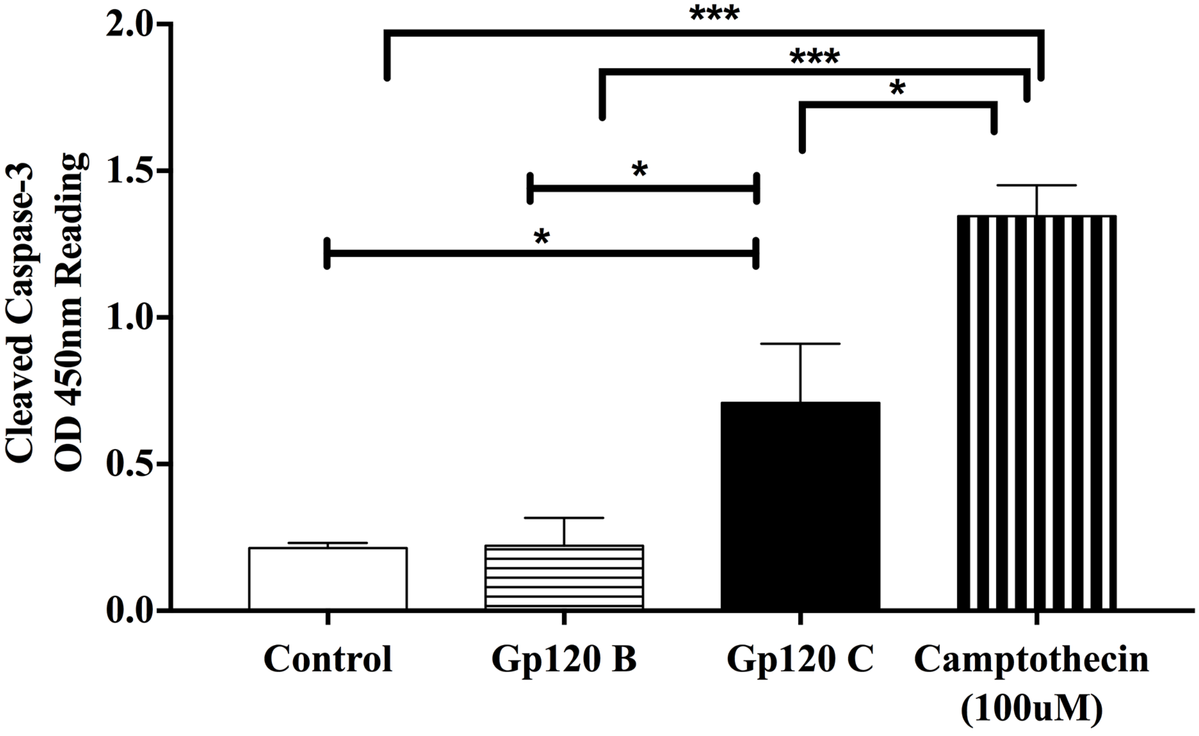

Supplementary Figure 3: HIV-1 gp120 clade C protein induces higher levels of cleaved caspase 3 protein expression in A172 cells. Cleaved caspase 3-protein expression by PathScan Sandwich ELISA was measured after HIV-1 gp120 clade B and C treatment. Mean ± SEM and statistical significance was determined using one-way ANOVA, $P \leq 0.05$ (N=3).

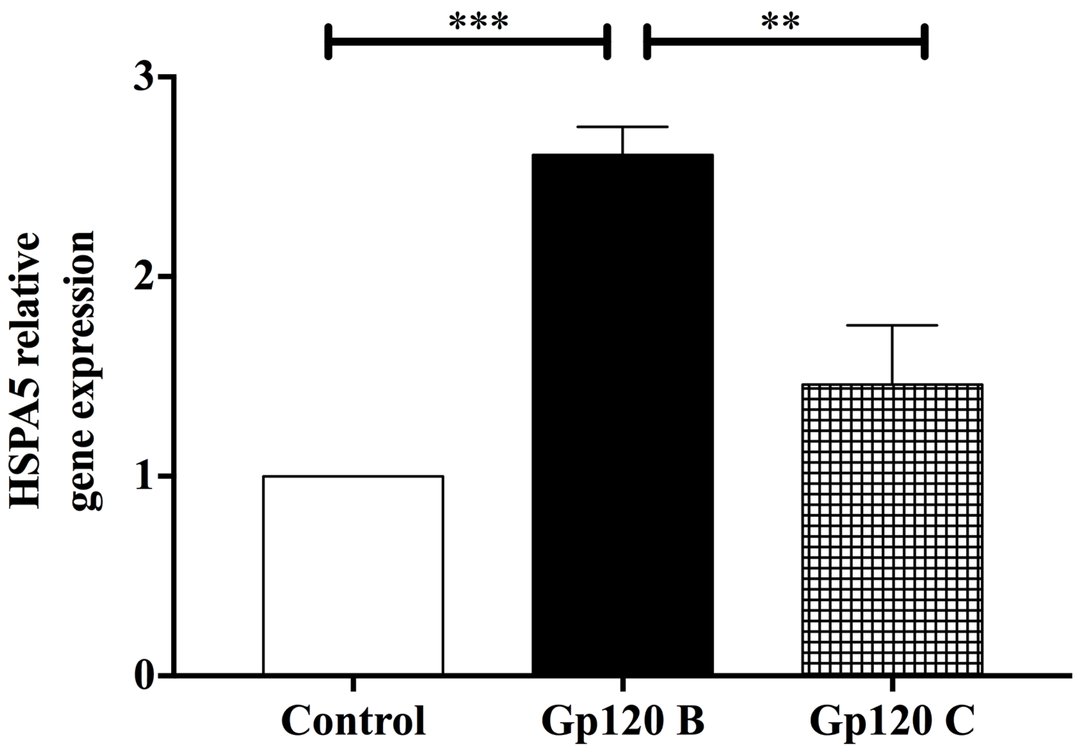

Supplementary Figure 4: HIV-1 gp120 clade B induces a protective response triggered by GRP78 Unfolded Protein Response marker. GRP78, an UPR key protein marker, was measured after HIV-1 gp120 clade B and C glioma treatment. GRP78 (also known as *HSPA5)* relative gene expression by qRT-PCR analysis was measured. qRT-PCR was normalized with GAPDH as housekeeping gene. Mean ± SEM and statistical significance was determined using one-way ANOVA, $P \leq 0.05$ (N=3).

Supplementary Table 1: Additional differentially expressed proteins induced by HIV-1 gp120 clade B in astrocytoma. Classification of differentially expressed proteins were distributed according to their biological function and analyzed by GSEA. Gene ontology identification (GO.ID), Sum of identified peptides (Σ# Peptides), Sum of the numbers of peptide spectrum matches (Σ# PSM's), Identified amino acid numbers (# AA's), Molecular weight (MW, kDa), Calculated isoelectric point (pH, calc. PI) and adjusted P-value as false discovery rate (FDR). Proteins with a FDR ≤ 0.025 were selected as significant for HIV-1 gp120 clade B treated astrocytoma cells.

See Supplementary File 1

Supplementary Table 2: Additional differentially expressed proteins induced by HIV-1 gp120 clade C in astrocytoma. Classification of differentially expressed proteins were distributed according to their biological function and analyzed by GSEA. Gene ontology identification (GO.ID), Sum of identified peptides (Σ# Peptides), Sum of the numbers of peptide spectrum matches (Σ# PSM's), Identified amino acid numbers (# AA's), Molecular weight (MW, kDa), Calculated isoelectric point (pH, calc. PI) and adjusted P-value as false discovery rate (FDR). Proteins with a FDR ≤ 0.025 were selected as significant for HIV-1 gp120 clade C treated astrocytoma cells.

See Supplementary File 2
