# Supplementary material for: HIV-1 Gp120 clade B/C induces a GRP78 driven cytoprotective mechanism in astrocytoma
Source: Oncotarget. 2017 Jul 22;8(40):68415–38. doi: 10.18632/oncotarget.19474 (PMC5620267; doi:10.18632/oncotarget.19474)
Supplement: Supplementary file 2 [file oncotarget-08-68415-s002.docx]

| **Gp120 B induced proteins** | | | | | | | | | |
| --- | --- | --- | --- | --- | --- | --- | --- | --- | --- |
| **GO.ID [a]** | **GSEA assigned biological process** | **Σ# Peptides [b]** | **Σ# PSM's [c]** | **AA's # [d]** | **MW [e]** | **calc. PI [f]** | **Adjusted P-value (FDR) [g]** | **Gene** | **Description** |
| **Translation/transcription** | | | | | | | | | |
| CORUM:305 | 40S ribosomal subunit, cytoplasmic | 1 | 1 | 121 | 13.7 | 5.81 | 0.002 | RPS13 | 40S ribosomal protein S13 |
|  |  | 1 | 2 | 196 | 22.2 | 8.53 |  | RPS15 | 40S ribosomal protein S15 |
|  |  | 1 | 1 | 63 | 7.1 | 10.26 |  | RPS2 | 40S ribosomal protein S2 |
|  |  | 1 | 3 | 165 | 18.9 | 10.15 |  | RPS10 | 40S ribosomal protein S10 |
| GO:0070937 | CRD-mediated mRNA stability complex | 1 | 3 | 599 | 67.4 | 5.82 | 0.004 | DHX9 | ATP-dependent RNA helicase A |
| REAC:429947 | deadenylation of mRNA | 1 | 2 | 108 | 12.2 | 8.46 | 0.000 | PABPC1 | Polyadenylate-binding protein 1 |
|  |  | 2 | 4 | 1403 | 154.7 | 5.16 |  | EIF4G1 | Eukaryotic translation initiation factor 4 gamma 1 |
|  |  | 2 | 6 | 200 | 23.2 | 5.69 |  | EIF4A1 | Eukaryotic initiation factor 4A-I |
| REAC:156842 | eukaryotic Translation Elongation | 2 | 5 | 206 | 24.1 | 7.05 | 0.000 | EEF1G | Elongation factor 1-gamma |
| GO:0016281 | eukaryotic translation initiation factor 4F complex | 2 | 4 | 1403 | 154.7 | 5.16 | 0.001 | EIF4G1 | Eukaryotic translation initiation factor 4 gamma 1 |
|  |  | 2 | 6 | 200 | 23.2 | 5.69 |  | EIF4A1 | Eukaryotic initiation factor 4A-I |
| GO:0048255 | mRNA stabilization | 1 | 2 | 108 | 12.2 | 8.46 | 0.003 | PABPC1 | Polyadenylate-binding protein 1 |
| **Metabolism** | | | | | | | | | |
| GO:0016832 | aldehyde-lyase activity | 3 | 6 | 364 | 39.4 | 8.09 | 0.026 | ALDOA | Fructose-bisphosphate aldolase A |
| KEGG:01230 | biosynthesis of amino acids | 8 | 31 | 516 | 56.2 | 8.44 | 0.000 | PKM | Pyruvate kinase PKM |
|  |  | 1 | 3 | 161 | 17 | 5.6 |  | PGK1 | Phosphoglycerate kinase 1 |
|  |  | 3 | 6 | 364 | 39.4 | 8.09 |  | ALDOA | Fructose-bisphosphate aldolase A |
|  |  | 6 | 30 | 335 | 36 | 8.46 |  | GAPDH | Glyceraldehyde-3-phosphate dehydrogenase |
|  |  | 2 | 4 | 315 | 34.7 | 4.87 |  | ENO2 | Gamma-enolase |
| GO:0030388 | fructose 1,6-bisphosphate metabolic process | 3 | 6 | 364 | 39.4 | 8.09 | 0.022 | ALDOA | Fructose-bisphosphate aldolase A |
| GO:0006094 | gluconeogenesis | 1 | 3 | 296 | 30.9 | 8.43 | 0.000 | MDH2 | Malate dehydrogenase, mitochondrial |
|  |  | 1 | 3 | 161 | 17 | 5.6 |  | PGK1 | Phosphoglycerate kinase 1 |
|  |  | 6 | 30 | 335 | 36 | 8.46 |  | GAPDH | Glyceraldehyde-3-phosphate dehydrogenase |
|  |  | 2 | 4 | 315 | 34.7 | 4.87 |  | ENO2 | Gamma-enolase |
| GO:0016259 | selenocysteine metabolic process | 1 | 1 | 121 | 13.7 | 5.81 | 0.000 | RPS13 | 40S ribosomal protein S13 |
|  |  | 1 | 2 | 196 | 22.2 | 8.53 |  | RPS15 | 40S ribosomal protein S15 |
|  |  | 1 | 1 | 63 | 7.1 | 10.26 |  | RPS2 | 40S ribosomal protein S2 |
|  |  | 1 | 3 | 165 | 18.9 | 10.15 |  | RPS10 | 40S ribosomal protein S10 |
|  |  | 3 | 7 | 414 | 46 | 11.36 |  | RPL4 | 60S ribosomal protein L4 |
| **Protein Transport/Degradation** | | | | | | | | | |
| REAC:264870 | caspase-mediated cleavage of cytoskeletal proteins | 2 | 6 | 4684 | 531.5 | 5.96 | 0.012 | PLEC | Plectin |
|  |  | 2 | 4 | 2452 | 282.1 | 5.34 |  | SPTAN1 | Spectrin alpha chain, non-erythrocytic 1 |
| GO:0070535 | histone H2A K63-linked ubiquitination | 1 | 1 | 241 | 28 | 5.29 | 0.029 | OTUB1 | Ubiquitin thioesterase OTUB1 |
| GO:0090004 | positive regulation of establishment of protein localization to plasma membrane | 1 | 2 | 1037 | 115.1 | 6.57 | 0.001 | ITGA3 | Integrin alpha-3 |
|  |  | 1 | 2 | 83 | 8.8 | 4.42 |  | GNB2L1 | Receptor of activated protein C kinase 1 |
| GO:0006611 | protein export from nucleus | 1 | 2 | 103 | 11.7 | 10.33 | 0.033 | RAN | GTP-binding nuclear protein Ran |
|  |  | 1 | 3 | 296 | 34.7 | 4.34 |  | CALR | Calreticulin |
| GO:0000060 | protein import into nucleus, translocation | 1 | 1 | 112 | 12 | 9.61 | 0.015 | PHB2 | Prohibitin-2 |
| **Cytoskeletal** | | | | | | | | | |
| CORUM:5615 | emerin complex 52 | 1 | 1 | 282 | 31.3 | 4.84 | 0.006 | C1QBP | Complement component 1 Q subcomponent-binding protein, mitochondrial |
|  |  | 3 | 9 | 233 | 26.5 | 4.87 |  | YWHAE | 14-3-3 protein epsilon |
|  |  | 1 | 2 | 450 | 50.1 | 6.64 |  | ILF3 | Interleukin enhancer-binding factor 3 |
| GO:0017166 | vinculin binding | 2 | 2 | 2429 | 257.9 | 6.49 | 0.022 | TLN1 | Talin-1 |
| **Calcium Signaling** | | | | | | | | | |
| GO:0005544 | calcium-dependent phospholipid binding | 2 | 4 | 204 | 22.7 | 5.53 | 0.002 | ANXA1, | Annexin A1 |
|  |  | 3 | 7 | 276 | 30.8 | 4.81 |  | ANXA5 | Annexin A5 |
|  |  | 4 | 10 | 339 | 38.6 | 7.75 |  | ANXA2 | Annexin A2 |
| GO:0048306 | calcium-dependent protein binding | 2 | 4 | 90 | 10.2 | 5.48 | 0.002 | S100A6 | Protein S100-A6 |
| REAC:901042 | calnexin/calreticulin cycle | 1 | 3 | 279 | 31.9 | 7.37 | 0.014 | PDIA3 | Protein disulfide-isomerase A3 |
|  |  | 1 | 3 | 296 | 34.7 | 4.34 |  | CALR | Calreticulin |
| **Autophagy** | | | | | | | | | |
| GO:0061684 | chaperone-mediated autophagy | 1 | 3 | 363 | 39.8 | 5.67 | 0.030 | LAMP2 | Lysosome-associated membrane glycoprotein 2 |
| **Other** | | | | | | | | | |
| GO:0015186 | L-glutamine transmembrane transporter activity | 1 | 2 | 541 | 56.6 | 5.48 | 0.022 | SLC1A5 | Neutral amino acid transporter B(0) |
| GO:0042555 | MCM complex | 1 | 2 | 279 | 31.7 | 8.6 | 0.001 | MCM4 | DNA replication licensing factor MCM4 |
|  |  | 1 | 2 | 762 | 85.6 | 6.15 |  | MCM3 | DNA replication licensing factor MCM3 |
| GO:1902808 | positive regulation of cell cycle G1/S phase transition | 1 | 1 | 560 | 62.7 | 9.68 | 0.003 | CDC6 | Cell division control protein 6 homolog |
|  |  | 1 | 1 | 112 | 12 | 9.61 |  | PHB2 | Prohibitin-2 |
| GO:2000304 | positive regulation of ceramide biosynthetic process | 1 | 2 | 83 | 8.8 | 4.42 | 0.006 | GNB2L1 | Receptor of activated protein C kinase 1 |
| GO:0051901 | positive regulation of mitochondrial depolarization | 1 | 2 | 83 | 8.8 | 4.42 | 0.004 | GNB2L1 | Receptor of activated protein C kinase 1 |
| GO:2000508 | regulation of dendritic cell chemotaxis | 1 | 1 | 282 | 31.3 | 4.84 | 0.001 | C1QBP | Complement component 1 Q subcomponent-binding protein, mitochondrial |
|  |  | 1 | 3 | 296 | 34.7 | 4.34 |  | CALR | Calreticulin |
| [a] Gene ontology identification | | |  |  |  |  |  |  |  |
| [b] Sum of identified peptides | | |  |  |  |  |  |  |  |
| [c] Sum of the numbers of peptide spectrum matches | | | |  |  |  |  |  |  |
| [d] Identified aminoacid numbers | |  |  |  |  |  |  |  |  |
| [e] Molecular weight, kDa | |  |  |  |  |  |  |  |  |
| [f] Calculated isoelectric point, pH | |  |  |  |  |  |  |  |  |
| [g] Adjusted P-value as false discovery rate | | |  |  |  |  |  |  |  |
