# Supplementary material for: HIV-1 Gp120 clade B/C induces a GRP78 driven cytoprotective mechanism in astrocytoma
Source: Oncotarget. 2017 Jul 22;8(40):68415–38. doi: 10.18632/oncotarget.19474 (PMC5620267; doi:10.18632/oncotarget.19474)
Supplement: Supplementary file 3 [file oncotarget-08-68415-s003.docx]

| **Gp120 C induced proteins** | | | | | | | | | | | | |
| --- | --- | --- | --- | --- | --- | --- | --- | --- | --- | --- | --- | --- |
| **GO.ID [a]** | **GSEA assigned biological process** | **Σ# Peptides [b]** | | | **Σ# PSM's [c]** | | **AA's # [d]** | **MW [e]** | **calc. PI [f]** | **Adjusted P-value (FDR) [g]** | **Gene** | **Description** |
| **Cytoskeletal** | | | | | | | | | | | | |
| GO:0051015 | actin filament binding | 2 | | | 3 | | 472 | 52.2 | 7.44 | 0.013 | FSCN1 | Fascin |
|  |  | 3 | | | 9 | | 1960 | 226.4 | 5.6 |  | MYH9 | Myosin 9 |
|  |  | 4 | | | 16 | | 692 | 79.9 | 5.17 |  | ACTN4 | Alpha actinin 4 |
| GO:0030048 | actin filament-based movement | 10 | | | 55 | | 431 | 49.6 | 5.25 | 0.000 | VIM | Vimentin |
|  |  | 1 | | | 3 | | 103 | 11.7 | 4.41 |  | MYL6 | Myosin light polypeptide 6 |
|  |  | 3 | | | 9 | | 1960 | 226.4 | 5.6 |  | MYH9 | Myosin 9 |
|  |  | 4 | | | 16 | | 692 | 79.9 | 5.17 |  | ACTN4 | Alpha actinin 4 |
|  |  | 1 | | | 2 | | 158 | 18.6 | 4.74 |  | TPM3 | Tropomyosin alpha-3 chain |
| GO:0097517 | contractile actin filament bundle | 1 | | | 2 | | 158 | 18.6 | 4.74 | 0.001 | TPM3 | Tropomyosin alpha-3 chain |
| GO:0030864 | cortical actin cytoskeleton | 2 | | | 2 | | 222 | 24.9 | 5.71 | 0.000 | VCL | Vinculin |
|  |  | 1 | | | 1 | | 250 | 27.3 | 9.1 |  | SLC2A1 | Solute carrier family 2, facilitated glucose transporter member 1 |
|  |  | 4 | | | 12 | | 426 | 46.3 | 7.66 |  | EEF1A1 | Elongation factor 1-alpha 1 |
| GO:0030898 | actin-dependent ATPase activity | 1 | | | 3 | | 103 | 11.7 | 4.41 | 0.002 | MYL6 | Myosin light polypeptide 6 |
|  |  | 3 | | | 9 | | 1960 | 226.4 | 5.6 |  | MYH9 | Myosin 9 |
| CORUM:5608 | emerin architectural complex | 9 | | | 44 | | 375 | 41.7 | 5.48 | 0.009 | ACTB | Actin, cytoplasmic 1 |
|  |  | 2 | | | 5 | | 465 | 53.2 | 6.52 |  | LMNA | Prelamin-A/C |
| REAC:390450 | folding of actin by CCT/TriC | 1 | | | 2 | | 474 | 50.9 | 6.8 | 0.000 | CCT2 | T-complex protein 1 subunit beta |
|  |  | 1 | | | 2 | | 283 | 32 | 8.5 |  | CCT5 | T-complex protein 1 subunit epsilon |
| **Autophagy** | | | | | | | | | | | | |
| GO:0061684 | chaperone-mediated autophagy | 6 | | | 10 | | 732 | 84.6 | 5.02 | 0.000 | HSP90AA1 | Heat shock protein HSP 90-alpha |
|  |  | 2 | | | 4 | | 312 | 34.8 | 7.53 |  | HSPA8 | Heat shock cognate 71 kDa protein |
|  |  | 2 | | | 4 | | 407 | 47.3 | 5.33 |  | GFAP | Glial fibrillary acidic protein |
|  |  | 4 | | | 12 | | 426 | 46.3 | 7.66 |  | EEF1A1 | Elongation factor 1-alpha 1 |
| **Protein Transport/Degradation** | | | | | | | | | | | | |
| REAC:5607761 | dectin-1 mediated noncanonical NF-kB signaling | 1 | | | 4 | | 138 | 16 | 6.14 | 0.000 | PSMD6 | 26S proteasome non-ATPase regulatory subunit 6 |
| REAC:4641257 | degradation of AXIN | 1 | | | 1 | | 591 | 64.5 | 6.86 | 0.000 | PSMD2 | 26S proteasome non-ATPase regulatory subunit 2 |
| REAC:195253 | degradation of beta-catenin by the destruction complex | 1 | | | 3 | | 261 | 29.5 | 7.72 | 0.001 | PSMA4 | Proteasome subunit alpha type-4 |
| REAC:4641258 | degradation of DVL | 1 | | | 5 | | 85 | 9.3 | 4.41 | 0.000 | PSMA6 | Proteasome subunit alpha type-6 |
| REAC:5610780 | degradation of GLI1 by the proteasome | 1 | | | 2 | | 241 | 26.4 | 4.79 | 0.000 | PSMA5 | Proteasome subunit alpha type-5 |
| GO:0006607 | NLS-bearing protein import into nucleus | 1 | | | 3 | | 74 | 8.2 | 6.62 | 0.012 | IPO5 | Importin-5 |
|  |  | 1 | | | 2 | | 699 | 78.8 | 5.11 |  | TNPO2 | Transportin-2 |
| **Calcium Signaling** | | | | | | | | | | | | |
| GO:0014722 | regulation of skeletal muscle contraction by calcium ion signaling | 1 | | | 2 | | 208 | 23.7 | 7.17 | 0.048 | GSTO1 | Glutathione S-transferase omega-1 |
| **Translation/Transcription** | | | | | | | | | | | | |
| GO:0022625 | cytosolic large ribosomal subunit | 1 | | | 2 | | 64 | 7.3 | 11.65 | 0.009 | RPL6 | 60S ribosomal protein L6 |
|  |  | 1 | | | 1 | | 180 | 19.8 | 9.95 |  | RPL3 | 60S ribosomal protein L3 |
|  |  | 1 | | | 2 | | 115 | 11.7 | 4.54 |  | RPLP2 | 60S ribosomal protein L2 |
| REAC:72764 | eukaryotic Translation Termination | 1 | | | 3 | | 122 | 14 | 10.05 | 0.005 | RPS4X | 40S ribosomal protein S4, X isoform |
| GO:1901838 | positive regulation of transcription of nuclear large rRNA transcript from RNA polymerase I promoter | 1 | | | 3 | | 297 | 32.4 | 5.36 | 0.021 | NCL | Calpain-3 |
| GO:0045727 | positive regulation of translation | 1 | | | 3 | | 112 | 12.6 | 8.57 | 0.001 | HNRNPD | Heterogeneous nuclear ribonucleoprotein D0 |
|  |  | 4 | | | 12 | | 583 | 64.6 | 8.06 |  | EEF2 | Elongation factor 2 |
|  |  | 3 | | | 6 | | 265 | 29.4 | 4.61 |  | NPM1 | Nucleophosmin |
|  |  | 1 | | | 3 | | 122 | 14 | 10.05 |  | RPS4X | 40S ribosomal protein S4, X isoform |
| **Metabolism** | | | | | | | | | | | | |
| KEGG:01230 | biosynthesis of amino acids | 1 | | | 2 | | 210 | 22.7 | 5.2 | 0.004 | PFKP | ATP-dependent 6-phosphofructokinase |
|  |  | 8 | | | 23 | | 531 | 58 | 7.71 |  | PKM | Pyruvate kinase PKM |
|  |  | 3 | | | 13 | | 341 | 36.9 | 6.28 |  | ENO1 | Alpha-enolase |
|  |  | 1 | | | 1 | | 406 | 43.2 | 7.15 |  | PHGDH | D-3-phosphoglycerate dehydrogenase |
|  |  | 3 | | | 5 | | 213 | 22.9 | 6.92 |  | TPI1 | Triosephosphate isomerase |
| KEGG:00270 | cysteine and methionine metabolism | 2 | | | 3 | | 232 | 25.2 | 5.81 | 0.005 | LDHB | L-lactate dehydrogenase B chain |
|  |  | 5 | | | 14 | | 332 | 36.7 | 8.27 |  | LDHA | L-lactate dehydrogenase A chain |
| GO:0005984 | disaccharide metabolic process | 1 | | | 1 | | 250 | 27.3 | 9.1 | 0.013 | SLC2A1 | Solute carrier family 2, facilitated glucose transporter member 1 |
| GO:0030212 | hyaluronan metabolic process | 1 | | | 1 | | 181 | 19.6 | 4.55 | 0.026 | CD44 | CD44 antigen |
|  |  | 4 | | | 9 | | 1639 | 187.8 | 5.69 |  | CLTC | Clathrin heavy chain 1 |
| **Other** | | | | | | | | | | | | |
| REAC:977225 | amyloid fiber formation | 2 | | | 13 | | 126 | 13.9 | 10.32 | 0.020 | HIST2H2BE | Histone H2B type 2-E |
|  |  | 2 | | | 6 | | 103 | 11.4 | 11.36 |  | HIST4H4 | Histone H4 |
| GO:0055059 | asymmetric neuroblast division | 1 | | | 2 | | 200 | 22.5 | 8.38 | 0.025 | RAB10 | Ras-related protein Rab-10 |
| GO:0007569 | cell aging | 2 | | | 5 | | 465 | 53.2 | 6.52 | 0.007 | LMNA | Prelamin-A/C |
|  |  | 3 | | | 6 | | 265 | 29.4 | 4.61 |  | NPM1 | Nucleophosmin |
|  |  | 1 | | | 3 | | 115 | 12.5 | 7.88 |  | MIF | Macrophage migration inhibitory factor |
| GO:0030010 | establishment of cell polarity | 1 | | | 1 | | 99 | 10.4 | 4.22 | 0.000 | MAP4 | Microtubule-associated protein 4 |
|  |  | 2 | | | 3 | | 472 | 52.2 | 7.44 |  | FSCN1 | Fascin |
|  |  | 1 | | | 2 | | 200 | 22.5 | 8.38 |  | RAB10 | Ras-related protein Rab-10 |
|  |  | 1 | | | 3 | | 103 | 11.9 | 5.55 |  | RAP1B | Ras-related protein Rap-1b |
|  |  | 3 | | | 5 | | 577 | 67.8 | 6.4 |  | MSN | Moesin |
| GO:0030952 | establishment or maintenance of cytoskeleton polarity | 2 | | | 5 | | 465 | 53.2 | 6.52 | 0.020 | LMNA | Prelamin-A/C |
| REAC:428790 | facilitative Na+-independent glucose transporters | 1 | | | 1 | | 250 | 27.3 | 9.1 | 0.022 | SLC2A1 | Solute carrier family 2, facilitated glucose transporter member 1 |
| REAC:5625900 | RHO GTPases | 1 | | | 3 | | 103 | 11.7 | 4.41 | 0.009 | MYL6 | Myosin light polypeptide 6 |
|  |  | 3 | | | 9 | | 1960 | 226.4 | 5.6 |  | MYH9 | Myosin 9 |
| [a] Gene ontology identification | | | | |  | |  |  |  |  |  |  |
| [b] Sum of identified peptides | | | | |  | |  |  |  |  |  |  |
| [c] Sum of the numbers of peptide spectrum matches | | | | | | |  |  |  |  |  |  |
| [d] Identified aminoacid numbers | | | |  |  | |  |  |  |  |  |  |
| [e] Molecular weight, kDa | | | |  |  | |  |  |  |  |  |  |
| [f] Calculated isoelectric point, pH | | | |  |  | |  |  |  |  |  |  |
